# Loss of histone macroH2A1 in hepatocellular carcinoma cells promotes paracrine-mediated chemoresistance and CD4^+^CD25^+^FoxP3^+^ regulatory T cells activation

**DOI:** 10.7150/thno.35045

**Published:** 2020-01-01

**Authors:** Oriana Lo Re, Tommaso Mazza, Sebastiano Giallongo, Paola Sanna, Francesca Rappa, Tu Vinh Luong, Giovanni Li Volti, Adela Drovakova, Tania Roskams, Matthias Van Haele, Emmanuel Tsochatzis, Manlio Vinciguerra

**Affiliations:** 1 International Clinical Research Center, St' Anne's University Hospital, Brno, Czech Republic;; 2 Department of Biology, Faculty of Medicine, Masaryk University, Brno, Czech Republic;; 3 IRCCS Casa Sollievo della Sofferenza, Laboratory of Bioinformatics, San Giovanni Rotondo (FG), Italy;; 4 Department of Experimental Biomedicine and Clinical Neurosciences, University of Palermo, Palermo, Italy;; 5 Department of Histopathology, Royal Free London NHS Foundation Trust, London, United Kingdom;; 6 Department of Biomedical and Biotechnological Sciences, University of Catania, Catania, Italy;; 7 Institute of Biophysics, Academy of Sciences of the Czech Republic, Brno, Czech Republic;; 8 Department of Animal Physiology and Immunology, Institute of Experimental Biology, Masaryk University, Brno, Czech Republic;; 9 Translational Cell & Tissue Research Unit, Department of Imaging & Pathology, Katholieke Universiteit Leuven, Leuven, Belgium;; 10 UCL Institute for Liver and Digestive Health, Royal Free Hospital, London, United Kingdom.

**Keywords:** hepatocellular carcinoma, histone macroH2A1, adaptive immune system, chemoresistance.

## Abstract

**Rationale:** Loss of histone macroH2A1 induces appearance of cancer stem cells (CSCs)-like cells in hepatocellular carcinoma (HCC). How CSCs interact with the tumor microenvironment and the adaptive immune system is unclear.

**Methods:** We screened aggressive human HCC for macroH2A1 and CD44 CSC marker expression. We also knocked down (KD) macroH2A1 in HCC cells, and performed integrated transcriptomic and secretomic analyses.

**Results:** Human HCC showed low macroH2A1 and high CD44 expression compared to control tissues. MacroH2A1 KD CSC-like cells transferred paracrinally their chemoresistant properties to parental HCC cells. MacroH2A1 KD conditioned media transcriptionally reprogrammed parental HCC cells activated regulatory CD4^+^/CD25^+^/FoxP3^+^ T cells (Tregs).

**Conclusions:** Loss of macroH2A1 in HCC cells drives cancer stem-cell propagation and evasion from immune surveillance.

## Introduction

Hepatocellular carcinoma (HCC) is the second leading cause of cancer-related death worldwide 1. In advanced HCC, the liver maintains only a poor level of function, which limits patient eligibility for surgery. HCC tumors contain a population of cancer stem cells (CSCs) that are associated with metastatic potential and relapse 2. These CSCs can self-renew and differentiate to give rise to virtually all HCC cell types 2. Interestingly, while CSCs themselves do not rapidly proliferate, their progeny have a high proliferative capacity, and markedly augment the tumor mass. Although conventional chemotherapeutics suppress the proliferating cells that comprise the bulk of the tumor 3, CSCs are resilient to chemotherapy. Consequently, the gold standard HCC chemotherapeutic Sorafenib increases the average patient survival time by only a few months 4. It is thus imperative to develop therapies that specifically target CSCs to enhance patient survival and wellbeing.

HCC typically arises as a result of hepatic injury and inflammation. As such, the physiological defensive response towards HCC strongly relies on the adaptive immune system and its cellular constituents: macrophages, natural killer (NK) cells, plasma cells, NKT cells, regulatory T cells (Treg) and CD4^+^ and CD8^+^ T cells 5. The immune system triggers a potent anti-tumor response during HCC development: hepatic tumor progression markedly increases in T and B-cell-deficient Rag1^-/-^ mice upon diethylnitrosamine (DEN)-induced liver injury 6. Moreover, impaired immune surveillance of senescent hepatocytes induces HCC development in mice, indicating that senescence surveillance is important for tumor suppression in the liver 7, 8. CSC-specific avoidance of immune-cell detection and destruction might allow tumor relapse 9, 10; whether this is the case in HCC is currently unclear. Autocrine/paracrine non-cell-autonomous mechanisms govern the interplay between CSCs and neighboring cancer, immune and stromal cells and regulate CSC expansion and self-renewal in various cancers 11-14. As CSCs have an important role in mediating drug resistance following chemotherapy and in immune evasion, efforts should be made to identify the specific factors secreted by hepatic CSCs and determine their influence on neighboring cells and the signaling and epigenetic mechanisms that control their production.

We previously demonstrated a link between the epigenetic regulatory properties of the histone variant macroH2A1 and the emergence of CSCs in HCC 15, 16. MacroH2A1 is the largest histone variant in nature: it is composed of a domain that is 66% homologous to histone H2A, and a C-terminal linker that connects the histone fold domain to a macro domain. MacroH2A1 can either repress or activate transcription in response to growth signals 17. MacroH2A1 exists as two alternatively exon-spliced isoforms, macroH2A1.1 and macroH2A1.2 17. In several cancer types, evidence supports a tumor-suppressive role for macroH2A1.1, while the role of macroH2A1.2 is dependent on the specific cancer context 18. Histone variants have crucial roles in all DNA-template based processes, including transcriptional regulation and DNA repair and differ from their canonical (H2A, H2B, H3 and H4) counterparts in stability, DNA wrapping, and possession of specialized domains that regulate access to DNA 19. MacroH2A1 expression is typically low in poorly differentiated and aggressive HCC subtypes compared to well-differentiated HCC. MacroH2A1 knockdown in HCC cell lines transforms them into CSC-like cells, thus illustrating that absence of macroH2A1 mediates cellular stem-like features and tumorigenic potential 15, 16. MacroH2A1 expression has also been associated with the pathogenesis of multiple cancers 19.

Here, we demonstrate that loss of macroH2A1 expression, as observed in many HCC models, can reprogram parental HCC cells to a CSC-like chemoresistant phenotype in a paracrine manner. Our functional and immunological analyses on the macroH2A1-dependent CSC-like secretome suggest that this epigenetic pathway might provide an under-appreciated link between cancer stemness propagation in the tumor microenvironment (TME), and tumor-cell evasion from adaptive immune surveillance.

## Materials and Methods

### Cell cultures and treatments

The HepG2 and Huh-7 parental lines (ATCC) were cultured in DMEM (1X) supplemented with 10% fetal bovine serum (FBS) and 1% penicillin/streptomycin. Stable knock-down (KD) of macroH2A1 was achieved by lentiviral transduction 16. HepG2 or Huh-7 control cells were exposed or not to conditioned media (CM) from macroH2A1 KD cells for 24 h. Chemoresistance experiments were performed by incubating cells for 72 h with 1 μM Sorafenib (SIGMA, Germany), 2 μM Doxorubicin (Santa Cruz Biotechnology, USA) or DMSO. Pentose phosphate pathway (PPP) inhibition in Huh-7 cells was achieved by incubating cells for 24-48 h with 2.5-5 μM Physcion (Sigma, Germany). Cell proliferation was measured by MTT assay 20. To assess population doubling time of HepG2 and Huh‑7 cells, suspension of cells containing 300,000 cells/well were applied to 6‑well plates. Number of the cells was counted every day up to 3 days after plating.

### Metabolomics

The Huh-7 central carbon metabolism profile was studied using ultra-high performance liquid chromatography - mass spectrometry (UHPLC-MS) to evaluate potential differences in 64 metabolites between macroH2A1 KD and control cells (Supplemental Table 1). *Metabolite extraction:* Cell pellets were re-suspended in cold extraction solvents [methanol/ethanol (1/1, v/v)] spiked with metabolites not detected in un-spiked cell extracts (internal standards) and incubated at -20 °C for 1 h. The samples were then vortexed and centrifuged at 18,000 x g, at 4 °C for 5 min. Supernatants were collected and kept at 4 °C, while cell pellets were re-suspended again in cold extraction solvents and incubated at -20 °C for 1 h. Samples were vortexed and centrifuged at 18,000 x g, at 4°C for 5 min and the supernatants were collected and pooled with the previous supernatant samples. Supernatants were dried under vacuum, reconstituted in water and re-suspended with agitation for 15 min. The samples were then centrifuged at 18,000 x g for 5 min at 4 °C and transferred to vials for UHPLC-MS analysis. Two different quality control (QC) samples were used to assess the data quality: 1. a QC calibration sample to correct for the different response factors between and within batches; and 2. a QC validation sample to assess how well the data pre-processing procedure improved the data quality. Randomized sample injections were performed, with each of the QC calibration and validation extracts uniformly interspersed throughout the entire batch run.

*Data Pre-Processing:* All data were processed using the TargetLynx application manager for MassLynx 4.1 software (Waters Corp., Milford, USA). Data pre-processing generated a list of chromatographic peak areas for the metabolites detected in each sample injection. An approximated linear detection range was defined for each identified metabolite, assuming similar detector response levels for all metabolites belonging to a given chemical class represented by a single standard compound. Data normalization was performed as previously described 21. The ion intensities detected for each peak were normalized within each sample, to the sum of the peak intensities in that sample. There were no significant differences (HuH-7: t-test=0.1611) between the total intensities used for normalization of the groups compared in the study.

*Data Analysis:* Once normalized, the dimensionality of the complex data set was reduced to enable easy visualization of any metabolic clusters in the different sample groups. Data reduction was achieved by multivariate data analysis, including non-supervised principal components analysis (PCA) and/or supervised orthogonal partial least-squares to latent structures (OPLS) approaches 22. Univariate statistical analyses were also performed to calculate the group percentage changes and the unpaired Student's t-test p-value for the following comparison: HuH-7 KD vs. HuH-7 CTL.

### Immunoblotting and ELISA

RayBio® Human Biotin Label Based Antibody Arrays - Human L-507 Array, Membrane (AAH-BLM-1A-2, RayBio®, US) was used to analyze the supernatant (conditioned media) of Huh-7 cells (control or macroH2A1 KD), according to manufacturer's instructions. A Human Cytokines antibody array membrane (Abcam, Germany) was used to analyze the supernatant (conditioned media) of HepG2 cells (control or macroH2A1 KD), according to manufacturer's instructions (ab133997, Abcam, US). Detection of IL-6 and IL-8 levels in the culture media of Huh-7 cells was performed using Quantikine® kits (Bio-Techne R&D Systems s.r.o., Prague, Czech Republic), according to manufacturer's instructions.

Nuclei protein fractions from HepG2 and Huh-7 CTL cells were isolated as previously described 23, 24. Primary antibodies were obtained from Active Motif (macroH2A1.1 and macroH2A1.2) and Cell Signaling Technology (H2B).

### T-cell activation assay

Peripheral blood mononuclear cells (PBMC) were isolated from buffy coats of healthy volunteers (University Hospital Brno) by density gradient centrifugation using Ficoll. Cell pellets were re-suspended in PBS and centrifuged at 200 x *g* for 15 min at 20^o^C. Total T lymphocytes were isolated using the Pan T-cell isolation kit (Miltenyi Biotech, Germany), according to manufacturer's instructions. T cells fluorescently stained using CD4^+^-FITC and C25^+^-PE antibodies (Biosciences, Germany) were processed for analysis in a BD FACSCanto^TM^ II Flow Cytometer (Becton Dickinson, Germany).

### Treg suppression assay

Treg suppression assay was performed using a Treg Suppression Inspector assay, according to manufacturer's instructions (Miltenyi Biotech, Germany). CD4^+^/CD25^+^/FoxP3^+^ Tregs purified from fresh T cells from healthy donor blood and incubated with either CTL media or macroH2A1 KD media for the whole length of the assay, were used as suppressor cells, and the CD4^+^/CD25^-^ fraction was used as responder cells. To setup the assay, CD4^+^ /CD25^+^/FoxP3^+^ were cultured with CD4^+^/CD25^-^ T cells at increasing ratios (1:0, 1:1, 1:2, 1:4, 1:8). As a control, CD4^+^/CD25^-^ responder cells were cultured alone. A total of 5×10^5^ CD4^+^/CD25^-^ responder cells labeled with CFSE (Sigma, Germany) were co-cultured with 5×10^5^ CD4^+^/CD25^+^/FoxP3^+^ Tregs in 48-well plates in a volume of 1 ml/well of TexMACS medium (Miltenyi Biotech, Germany) supplemented with 100 U/ml of penicillin/streptomycin (ThermoFisher Scientific, Waltham, USA), and Treg Suppression Inspector, composed of anti-biotin MACSiBead™ particles preloaded with biotinylated anti-CD2, anti-CD3, and anti-CD28 antibodies (Miltenyi Biotech, Germany) for four days. Cells were then analyzed in a BD FACSCantoTM II Flow Cytometer (Becton Dickinson, Germany).

### RNA-Seq and bioinformatic analyses

Total RNA was extracted from control and macroH2A1 KD Huh-7 cells using TRIzol Reagent (ThermoFisher Scientific, Waltham, USA). Indexed libraries were prepared from 250 ng purified RNA using the NEBNext Ultra II Directional RNA Library preparation kit with polyA selection module (New England Biolabs, Ipswich, USA). Libraries and input RNA samples were quality checked using Fragment Analyzer (Advanced Analytical, Ankeny, US). The libraries were pooled so that each index-tagged sample was present in equimolar amounts. The pooled samples then underwent cluster generation and sequencing using an Illumina NextSeq 500 (Illumina, San Diego, US) in a 2 x 75 paired-end format. Short reads were aligned against the hg19 genome assembly using STAR (ver. 2.6.1a). Piled-up reads were counted with *htseq-count* 25. Read-count normalization and comparisons were performed using the *edgeR* R package. Genes were considered differentially expressed between groups if their expression values significantly differed by ≥2 folds. Statistical differences in gene expression were assessed by the ANOVA test. Correction for multiple test was achieved by the Benjamini-Hochberg procedure. The significance threshold was set to 0.05. Functional and pathway enrichment analyses were performed using the Ingenuity Pathway Analysis (IPA, Milan, Italy) software tool. The RNASeq profiling data have been deposited to the GEO database, with accession number GSE131680.

### Immunohistochemistry

The Institutional Ethical Review Committee of the University Hospitals of Leuven approved the use of human HCC samples in this study. A total of 18 formalin-fixed paraffin-embedded (FFPE) liver biopsies from patients with HCC, treated at the University Hospitals of Leuven between 2003 and 2008, were included in this study. Patient clinicopathological features and immunohistochemistry methods were previously described 20, 26. A further 20 samples from explanted livers of patients undergoing liver transplantation for HCC at the Royal Free Hospital (London, UK) were included (Supplemental Table 2). All patients signed informed consent forms for the use of the explanted liver tissue for research as part of the standard consent process for liver transplantation. Immunohistochemical staining on samples from UK human cases of encapsulated or non-encapsulated HCC was conducted according to previously described protocols 24, 26-28, using Abcam antibodies (Cambridge, UK) against macroH2A1, macroH2A1.1, macroH2A1.2, p16, CD44 or CD4^+^. Immunohistochemical analysis of the cases from the University Hospitals of Leuven was performed as previously described 29. Semi-quantitative scoring of staining was performed in blind by two expert anatomo-pathologists (MVH and FR), as it was previously described 24, 26-28.

### Statistical analyses

The data represent the means ± s.e.m. Comparisons between groups were made using the Student's t-test or the Mann-Whitney U-test, as appropriate, using GraphPad Prism Software (version 5.00 for Windows, San Diego, CA, USA). A P-value ⩽0.05 was considered statistically significant.

## Results

### Human HCCs with stem-like characteristics express low levels of macroH2A1

High levels of macroH2A1 expression in human patients indicate differentiated HCC 23, 30.

**Figure 1:**
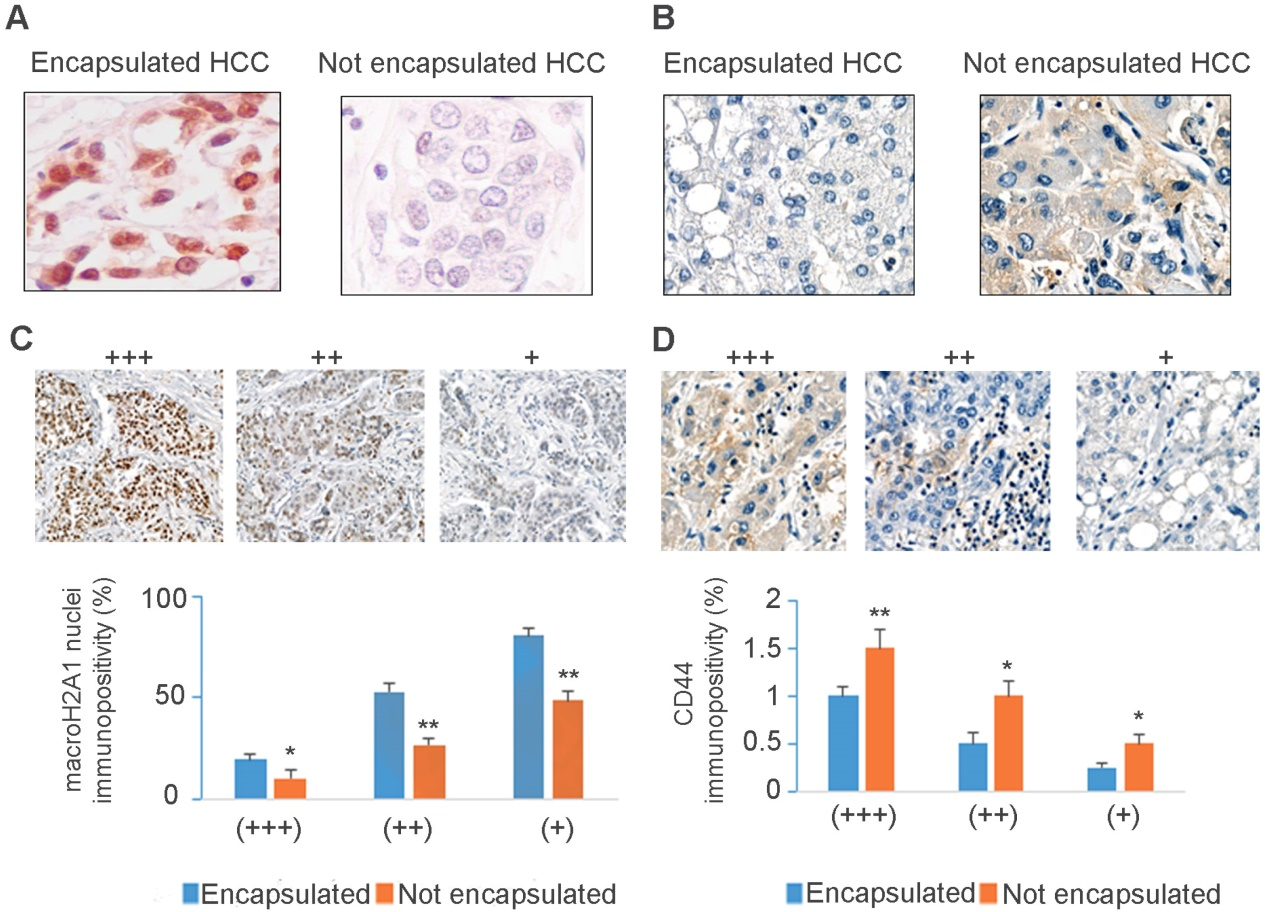
** MacroH2A1 and CD44 expression levels negatively correlate in encapsulated/non-encapsulated HCC from explanted livers of patients undergoing liver transplantation.** Representative immunohistochemical staining of macroH2A1 **(A)** or CD44 **(B)** in encapsulated versus non-encapsulated (metastatic) tumors. **(C, D)** Semi-quantitative scoring of cases as in A and B, based on high (+++), intermediate (++) and low (+) nuclear staining intensity, and the score of cells with the defined nuclear staining intensity from 0 (low) to 100 (high). N = 10 encapsulated tumors, N=10 non-encapsulated tumors. * p < 0.05; ** p < 0.01.

Conversely, macroH2A1 is weakly expressed in HCCs with a high level of CSCs (such as advanced-stage and sporadic fibrocellular HCC) compared to highly differentiated tumors 16. However, we did not previously consider a crucial prognostic factor, tumor encapsulation. Encapsulated HCCs exhibit a grossly distinct capsule-like fibrous tissue surrounding well-differentiated tumors, and are associated with a protracted clinical course and increased survival 31. We assessed macroH2A1 and CD44 expression by immunohistochemical labeling of HCC biopsies from patients with encapsulated or non-encapsulated tumors (n=10 per group) (Figure 1A and B) (Supplemental Table 2). As macroH2A1 staining was heterogeneous in both tumor groups, we applied a semi-quantitative scoring method as follows: high (+++), intermediate (++) and low (+) nuclear staining intensity (Figure 1C, upper panel). We combined this score with a score ranging from 0 to 100 to define the percentage of cells with defined nuclear staining intensity, in any randomly chosen field (Figure 1C, lower panel). The same scoring procedures were used to define CD44 staining.

Our analysis confirmed a significant down-regulation of macroH2A1 immunopositive cells in non-encapsulated compared to encapsulated HCC (Figure 1C), regardless of variations in intratissutal staining intensity. We also confirmed a negative correlation between CD44 and macroH2A1 expression: encapsulated tumors displayed weak or no CD44 staining, and non-encapsulated tumors showed strong CD44 staining (Figure 1B-D). In summary, down-regulation/loss of macroH2A1 histone is associated with human non-encapsulated HCC.

### MacroH2A1 knock-down (KD) HCC cells up-regulate glycolytic pathways

**Figure 2:**
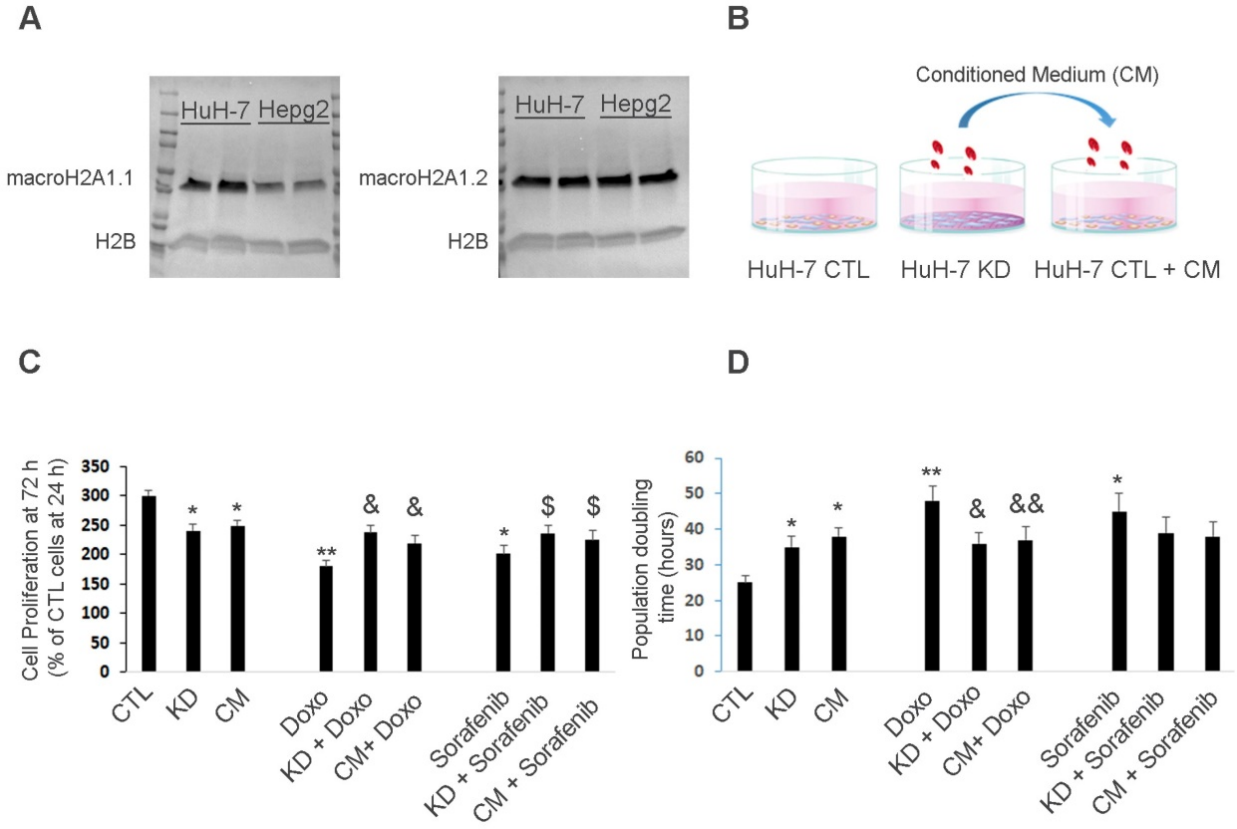
** Huh-7 macroH2A1 KD cells confer chemoresistance to parental cells in a paracrine manner. A.** Nuclear fractions isolated from CTL HepG2 or Huh-7 cells, were processed for immunoblotting with anti-macroH2A1.1, anti-macroH2A1.2 and anti-H2B antibodies. **B.** Three experimental conditions: control (CTL), KD and CTL cells plus KD conditioned medium (CM). **C.** MTT assay in CTL, KD or CTL + CM cells incubated with or without vehicle (DMSO), 2 µM Doxorubicin (Doxo) or 1 µM Sorafenib for 72 h. Data represent the mean cell proliferation ± s.d. relative to CTL cells at 24 h. N=4. **D.** Population doubling time in CTL, KD or CTL + CM cells incubated with or without vehicle (DMSO), 2 µM Doxorubicin (Doxo) or 1 µM Sorafenib for 72 h. Data represent the mean cell proliferation ± s.d. N=3.*P < 0.05, ** P < 0.01 relative to CTL; ^&^ P < 0.05,^& &^ P < 0.01 relative to Doxo; ^$^ P < 0.05,^$ $^ P < 0.01 relative to Sorafenib.

We previously showed that macroH2A1 KD induces CSC-like cellular architecture, morphology and behavior in HepG2 and Huh-7 HCC cell lines 16. Central carbon cell metabolism (CCM) supports multiple physiological processes including biosynthesis, amino acid homeostasis, epigenetic maintenance, and redox defense 32. CCM converts carbohydrates into metabolic precursors via three main pathways: glycolysis, the PPP and the citric acid cycle. CSCs are able to reprogram CCM to cater disease needs 33. Huh-7 is more aggressive than HepG2 cells, *in vitro* and *in vivo*, and the two cell lines have a different genetic make-up 30, 34-36. Moreover, control HepG2 and Huh-7 cells display similar levels of macroH2A1.2 isoform protein expression; however Huh-7 cells display higher macroH2A1.1 basal protein expression compared to HepG2 (Figure 2A). We have previously shown that macroH2A1 KD in HepG2 cells leads mainly to lipid metabolism reprogramming - with massive Acetyl-coA accumulation - and to a lesser extent to activation of glycolytic pathways 15. Here we aimed to understand the central CCM profiles required to target CSCs using Huh-7 cells, which is instead one of the most aggressive HCC cell lines 30, 34-36. We analyzed the endogenous metabolic profiles of control and macroH2A1 KD Huh-7 cells using an optimized UHPLC-MS platform that can profile 64 metabolites involved in CCM (Table S1). We initially performed an Orthogonal Partial Least Squares Classification for High Dimensional Data (OPLS-DA) analysis of control (n=7) and KD (n=7) cells to visualize any differences between the two groups (Figure S1A). This analysis identified a clear clustering of samples according to the experimental group (Figure S1A). We then generated a loading scatter plot based on the OPLS-DA analysis to identify the variables underlying this differential clustering. The metabolites lying away from the plot origin (having the strongest impact on the model) included nucleotides, redox/electron carriers and carbohydrates (Figure S1B).

We then conducted a univariate data analysis to calculate and compare (Student's t-test) the group percentage change and fold change for each individual metabolite between control and KD Huh-7 cells. Significant differences were found between 17 of the 64 tested metabolites in KD cells compared to control Huh-7 cells. Specifically, the levels of redox electron carriers (NADP, NADPH) and nucleotides (including ATP) were increased in Huh-7 KD cells compared to control cells (Figure S1C-D). These changes are consistent with increased activation of glycolytic branches that generate reduced forms of electron carriers and ATP in CSCs, and hyper-activation of the PPP 37 (Figure S2). Consistent with PPP activation, the fructose-6-phosphate (F6P)/glucose-6-phosphate (G6P) ratio was significantly decreased in HuH-7 KD compared to control cells (Figure S1C-D). In summary, these data suggest that macroH2A1 KD-induced CSC-like cells display activated glycolytic branches, particularly in the PPP.

### MacroH2A1 KD HCC cells can confer chemoresistance to parental HCC cells in a paracrine manner

MacroH2A1 modulates the transcriptional activity of cytokine/chemokine genes such as IL-6, IL-8, CXCL1 and CXCL6 38, which fine-tune CSC homeostasis 39. As modulated glycolytic activity is linked to altered cytokine production in immune cells 40, we hypothesized that the pro-glycolytic metabolic profile of macroH2A1 KD Huh-7 cells would change the cancer-associated secretome and affect the phenotype of neighboring parental HCC cells. To test our paracrine hypothesis, we exposed control Huh-7 cells to the CM of macroH2A1 KD cells for 72 h (Figure 2B). Consistent with previous studies 16, cell proliferation was lower in macroH2A1 KD and macroH2A1 KD CM-exposed Huh-7 cells, compared to control, non-exposed cells (Figure 2C).

Doxorubicin (an anthracycline antibiotic) and Sorafenib (a multi-tyrosine kinase inhibitor) are commonly used chemotherapeutics for advanced HCC. We incubated control, macroH2A1-KD, and CM-exposed Huh-7 cells with 2 µM Doxorubicin or 1 µM Sorafenib for 72 h, and found that both macroH2A1 KD and CM from KD cells could confer resistance to drug-induced cell proliferation inhibition (Figure 2C). Population doubling analyses mirrored the cell proliferation analyses (Figure 2D). However, when we incubated control, macroH2A1-KD, and CM-exposed HepG2 cells with 2 µM Doxorubicin or 1 µM Sorafenib for 72 h, we found that the CM from macroH2A1 KD was not able to confer resistance to drug-induced cell proliferation inhibition in this cell line (Figure S3A, B). These data support that macroH2A1-dependent chemoresistance can be conferred in a paracrine fashion in Huh-7 cells but not in HepG2 cells.

### MacroH2A1 KD HCC cells can transcriptionally reprogram parental HCC cells and produce a cytokine-depleted medium

**Figure 3:**
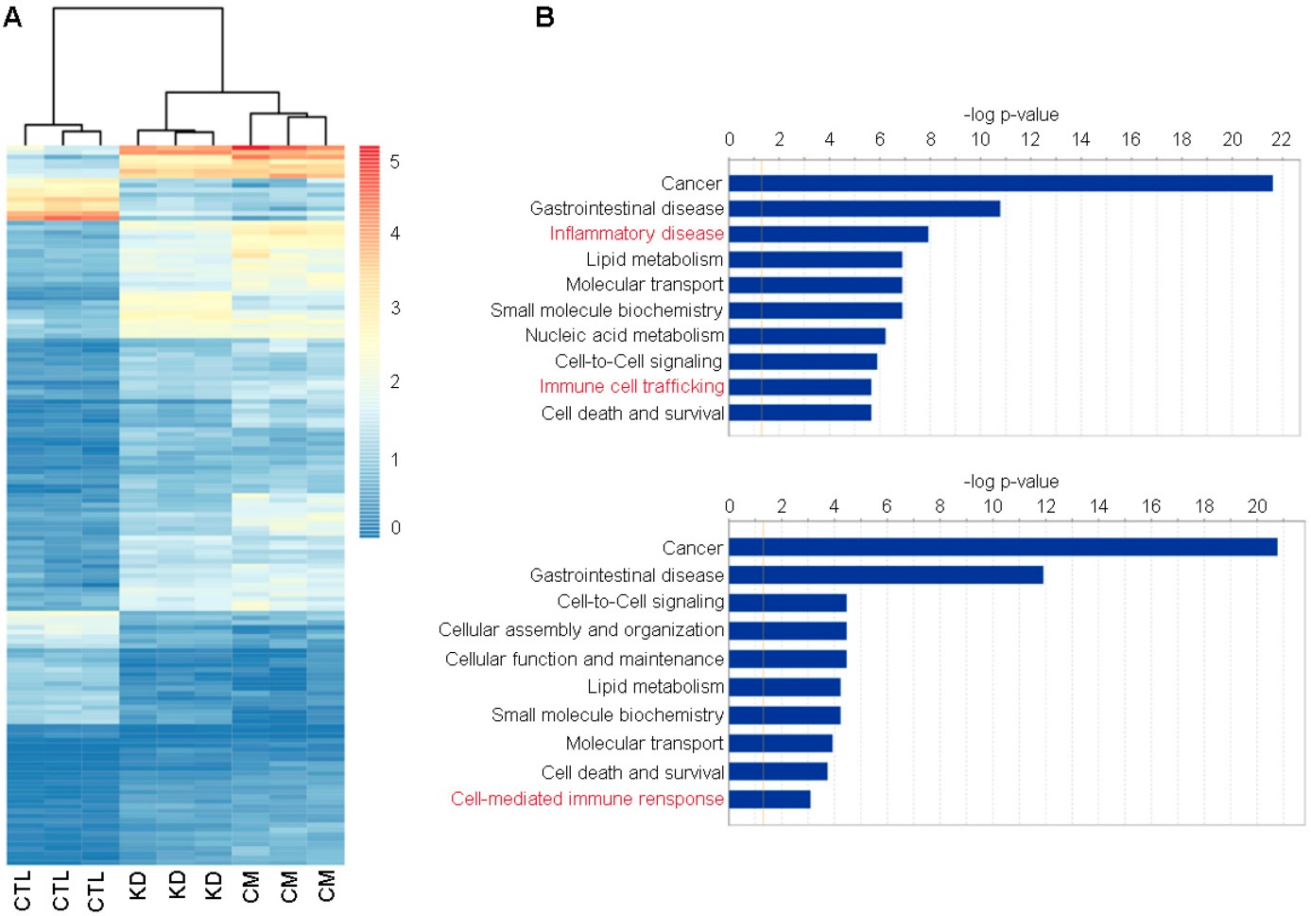
** Functional enrichment of commonly differentially expressed genes in control (CTL), macroH2A1 knockdown (KD) and conditioned media (CM) Huh-7 cells. A.** Heatmap of counts per million (cpm) values of commonly differentially expressed genes in KD vs CTL and CM vs CTL. **B.** Plot showing the enrichment results of KD vs CTL (top) and CM vs CTL (bottom) comparisons. Bar lengths represent the -log(p-value) of the enriched functions. The orange line indicates the statistically significant threshold. Asterisks denote functions implicated in inflammatory and immune responses.

We next used RNA-Seq to gain deep mechanistic insight into the distinct functions of macroH2A1 KD and (CM-)exposed Huh-7 cells. A heatmap drawn for the 202 commonly differentially expressed genes between the three groups revealed a similar transcriptomic profile between KD and CM, compared to the control condition (Figure 3A).

The transcriptomic profile similarity between CM and KD in comparison to CTL was also confirmed by measuring the Euclidean distance between centroids of clusters obtained by K-means algorithm (*data not shown*, 41).

Assessment of differentially expressed genes for Huh-7 macroH2A1 KD or Huh-7 CM KD versus CTL showed no transcriptional overlap between the different Huh-7 cell lines. 783 and 987 genes were significantly and differentially expressed (|FC|≥2, adj. p-value ≤0.05) over a total number of 26,439 screened genes, in macroH2A1 KD or CM KD versus control cells, respectively (Figure S4A, B). Interestingly, the significantly enriched genes, over-represented in KD and CM-exposed cells compared to control cells, were implicated in a number of functions and diseases that were also shared between CM *versus* CTL comparisons (Figure 3B). Specifically, Ingenuity pathway analysis (IPA) identified categories of cancer, gastrointestinal diseases, lipid metabolism, cell-to-cell signaling, nucleic acid metabolism, cell death & survival and others, in common between KD *versus* CTL and CM *versus* CTL (Figure 3B). These data support a paracrine modulation of gene expression by macroH2A1 KD in HCC cells.

**Figure 4:**
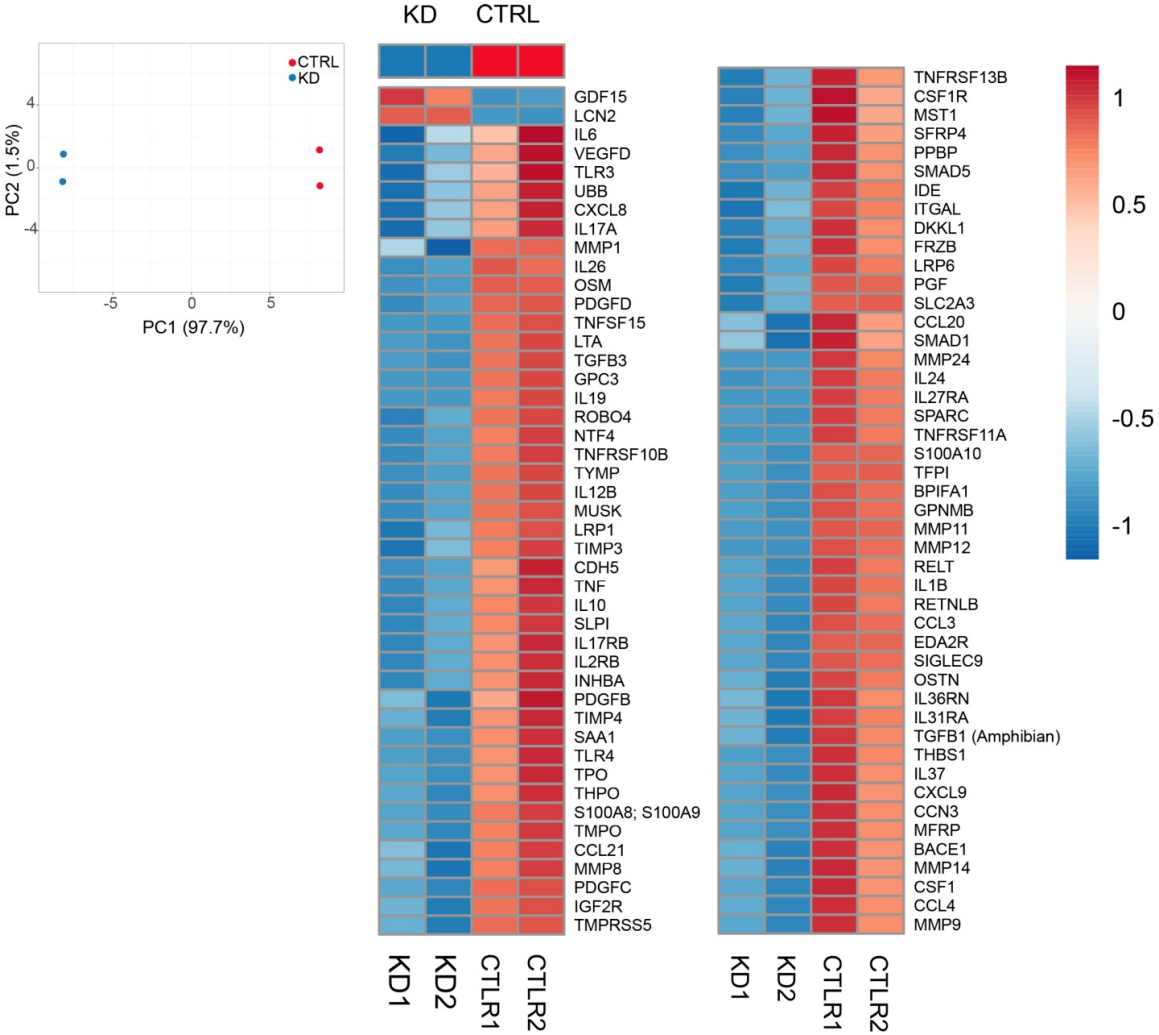
** Secretome analysis.** A. Principal Component Analysis applied to expression profiles of 507 human cytokines, chemokines, adipokine, growth factors, angiogenic factors, proteases, soluble receptors and soluble adhesion molecules in KD and CTRL cells. B. Heatmap representation of 94 differentially expressed molecules between KD (blue group) and CTRL cells (red group). Expression values were scaled between -1 and 1, where positive numbers were represented in tones of red, blue otherwise.

**Figure 5:**
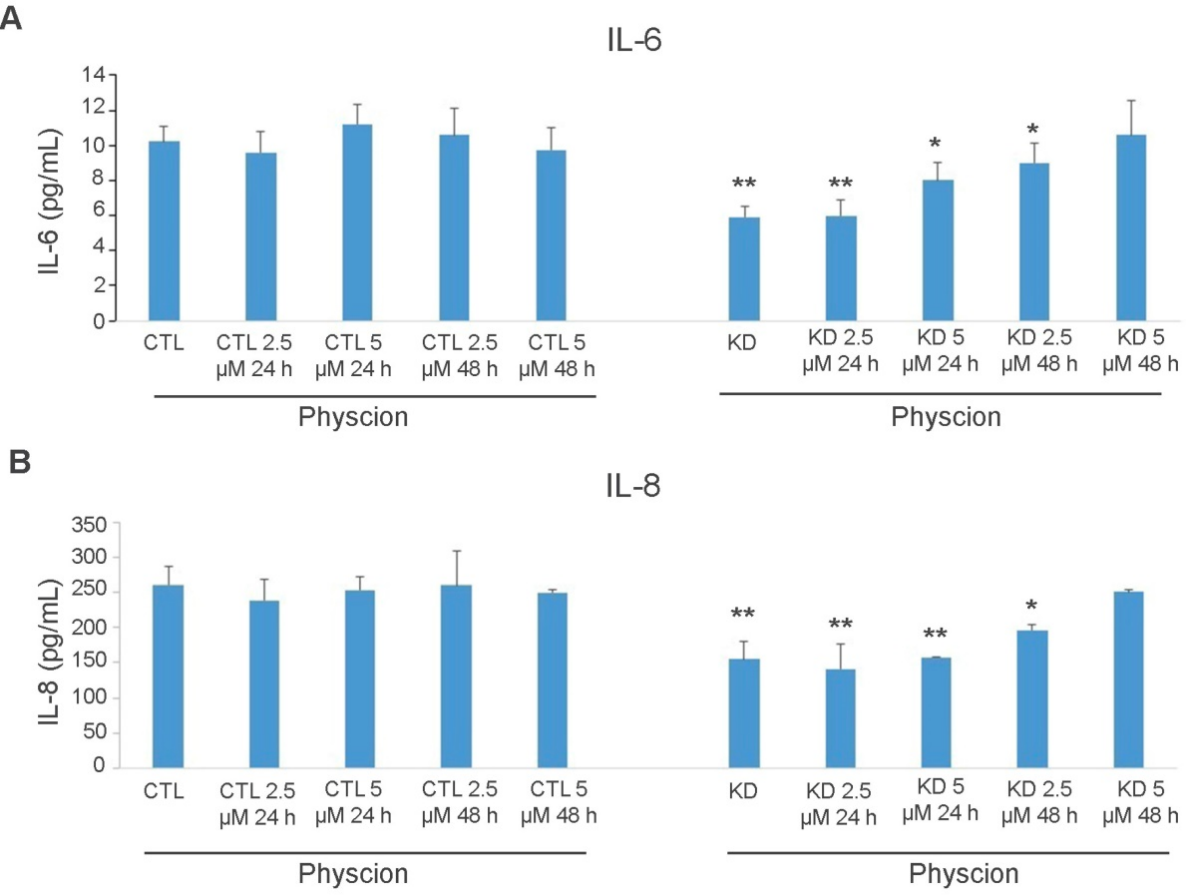
** ELISA analysis of cytokine levels in Huh-7 macroH2A1 knockdown (KD) compared to control (CTL) cells. A-B.** CTL and KD cultured cells were treated with Physcion (2.5 and 5 µM) for 24 h or 48 h or untreated. IL-6 (A) or IL-8 (B) or levels (in pg/ml) were measured in the cell supernatants using commercial ELISAs. N = 3. *p < 0.05, **p < 0.01 compared to control untreated cells.

Although Huh-7 cells were not exposed to any treatment other than the CM from KD cells, IPA revealed an enrichment in the categories of inflammatory disease, immune-cell trafficking (KD *versus* CTL), and cell mediated-immune responses (CM *versus* CTL) (Figure S5). As inflammatory and immune responses are chiefly mediated via secreted factors, we performed a quantitative analysis of a commercial comprehensive panel of 507 human cytokines, chemokines, adipokine, growth factors, angiogenic factors, proteases, soluble receptors and soluble adhesion molecules, detectable in Huh-7 CTL and macroH2A1 KD cell supernatants. Figure 4A shows a principal component analysis (PCA) which identified a clear clustering of samples according to the experimental group. Setting a fold-change barrier to 1.2, and the significance level to ≤0.05 (Benjamini-Hochberg adjusted p-value), we detected 94 differentially expressed factors (Figure 4B). Stunningly, with the exception of GDF15 and Lipocalin 2 (LCN2) that were upregulated, the remaining secreted factors were underrepresented in the supernatant of macroH2A1 KD Huh-7 cells (Figure 4B). In contrast, no changes were observed in HepG2 cells, between CTL and macroH2A1 KD, upon quantitative analysis of a panel of most representative 48 human cytokine/chemokines (Figure S6). To ascertain whether the decreased levels of secreted factors in the macroH2A1 KD Huh-7 secretome were linked to their reprogrammed metabolism, namely PPP activation (Figure 2, Figure S2), we preincubated control and macroH2A1 KD cells for 24-48 h with 2.5-5 μM Physcion — an effective inhibitor of the third enzyme of the PPP 42. We then assessed the levels of the two key pro-inflammatory cytokines, IL-6 and IL-8 (CXCL8) in the supernatants by ELISA (Figure 5). We confirmed downregulation of IL-6 and IL-8 protein levels in the macroH2A1 KD supernatant compared to control cells (Figure 5A, B). Physcion restored IL-6 and IL-8 levels in the KD cell supernatant upon 48 h incubation with a 5 μM dose, whereas no effect was seen in control cells (Figure 5A, B). These findings suggest that macroH2A1-dependent decreased IL-6/IL-8 secretion may be dependent on PPP activation.

### HCC harboring low macroH2A1 expression displays decreased CD4(+) lymphocyte infiltration

**Figure 6:**
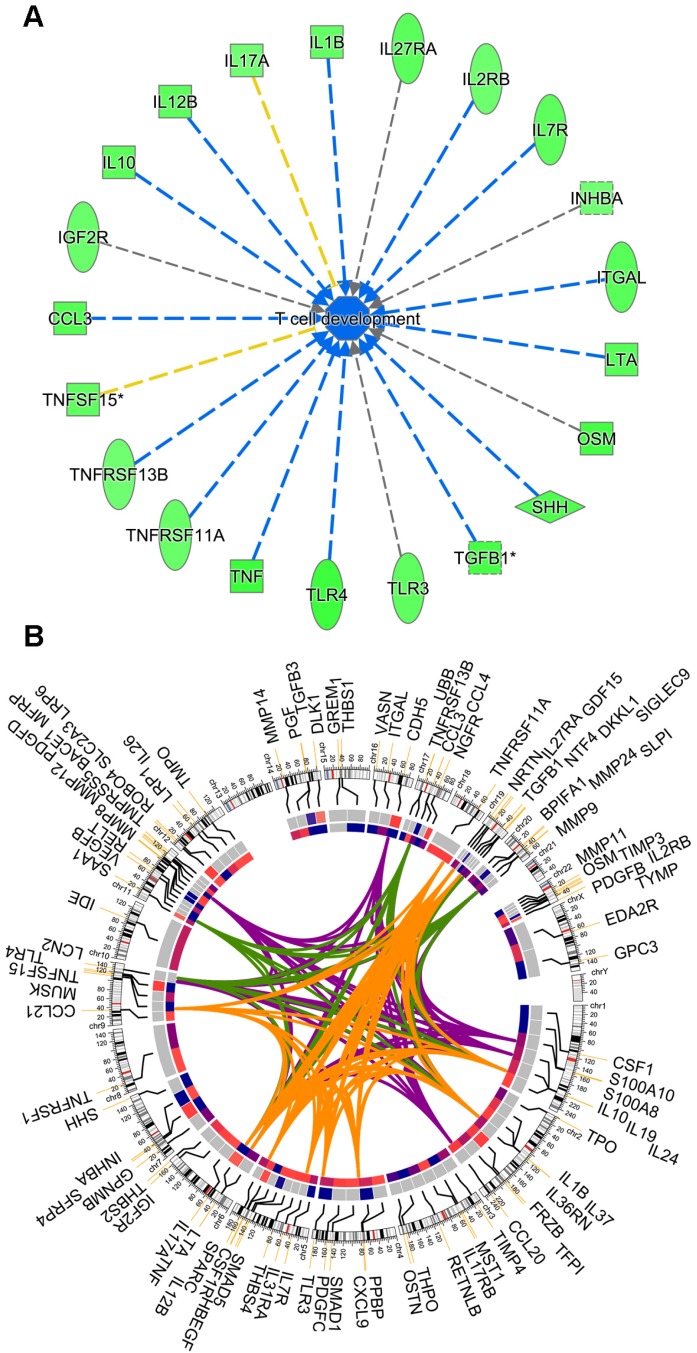
** (A)** Activity inference of the *T cell development/maturation* biological process by 21 out of the 94 differentially expressed molecules between KD and CTRL cells. The process is colored in blue, since its activity is predicted by IPA to be globally reduced in KD cells. Edges are colored (i) in blue, when the fold-regulation of the participating molecules to the process is coherent with the activity inference, (ii) in yellow, when there is inconsistency and (iii) gray when it is not possible to infer the kind of participation (activation, arrow or inhibition, dead-end arrow) of a molecule to the overall process. **(B)** Circos plot representing (from outer to the innermost circle) the 94 molecules, their chromosomal location, their fold-regulation values in the RNA-Seq experiment, and their fold-regulation values in the 94 panel of 507 secreted molecules. Genes were linked by colored bands, if belonging to the same biological process: orange (T cell response), green (activation of neutrophils) and violet (recruitment of neutrophils).

The overall cytokine/chemokine depleted medium of macroH2A1 KD CSC-like cells led us to hypothesize that loss of macroH2A1 might trigger pleiotropic effects that help CSCs evade the defensive immune response in a paracrine manner. Consistently, running IPA on the Huh-7 secretome (Figure 4), we inferred that this cytokine depletion would inhibit the process of T cell development/maturation (Figure 6A). Surprisingly, the changes in the secretome in Huh-7 macroH2A1 KD cells is largely independent on macroH2A1-dependent transcriptional regulation: through integrated transcriptomic and secretomic analyses we detected significant concordance between differentially expressed genes and differential expression of the corresponding secreted factors only in 10 over 94 factors (CCL3, CXCL9, HBEGF, IL7R, INHBA, MMP12, MMP24, PDGFB, PDGFD, TGFB3, TLR3) (Circos plot, Figure 6B).

Local immune responses in patients with HCC are restrained by tumor and circulating CD4^+^/CD25^+^/FoxP3^+^ Tregs, which suppress effector CD4^+^ T-cell activity and proliferation. Tregs are the predominant cell-type involved in HCC immune escape 43. Consequently, compromised lymphocyte infiltrates (namely, tumor infiltrating lymphocytes; TILs) are observed in HCC 44. Histological examination of encapsulated and non-encapsulated human HCC did not reveal any differences in total lymphocyte infiltration, regardless of tumoral or peri-tumoral localization (Figure S7A).

**Figure 7:**
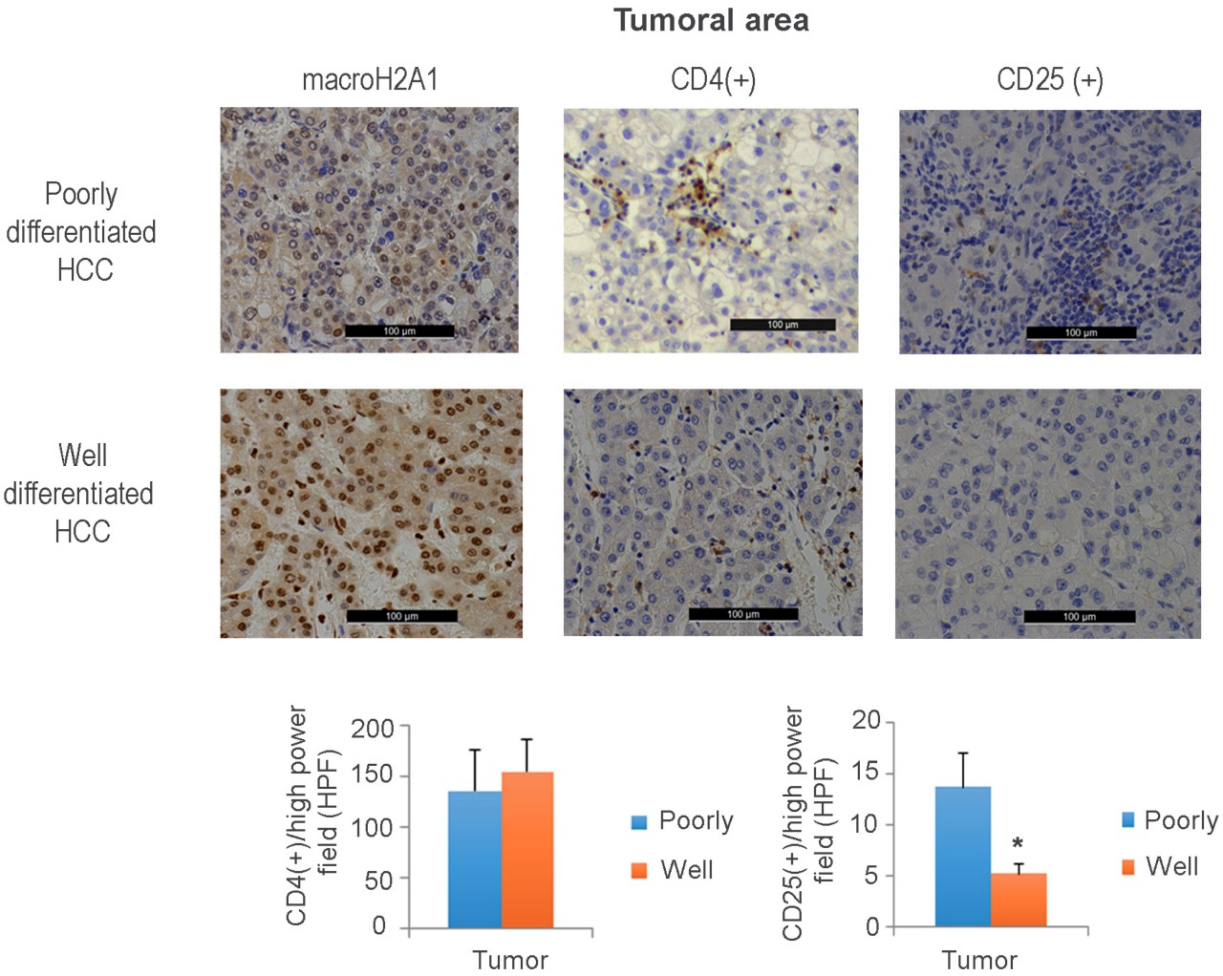
** Aggressive hepatocellular carcinoma (HCC) with reduced macroH2A1 expression displays increased CD25^+^infiltration.** Immunohistochemical staining for macroH2A1, CD4 and CD25 in poorly differentiated (n = 18) and well-differentiated (n = 16) HCC, from a previously characterized cohort 26. Magnification, 20 X. Black scale bars indicate 100 μM. The lower panels show the mean number of cells counted in 10 high-power fields (HPF) performed by a pathologist blinded to the HCC differentiation categories. *p < 0.01 compared to well differentiated.

We then examined whether reduced macroH2A levels correlate with a decreased frequency of CD4^+^ T cells specifically, or an increased presence of CD25^+^ TILS. Here, we used a previously described cohort 26 that we classified into two groups: patients with poorly differentiated HCC and low macroH2A1 expression; and patients with well-differentiated HCC and high macroH2A1 expression (Figure 7).

Immunohistochemical staining against CD25^+^ T cells revealed an increased tumoral presence in poorly differentiated versus well-differentiated tumors; the intra-tumoral CD4^+^ cell content was unchanged between the two HCC groups (Figure 7). Interestingly, a decreased presence of CD4^+^ cells was observed in the peritumoral area of poorly versus well-differentiated HCC (Figure S7B). These data demonstrate that aggressive HCC expressing low levels of macroH2A1 exhibits high levels of intra-tissue TILS expressing the CD25^+^ Treg marker and a decreased adaptive immune response.

**Figure 8:**
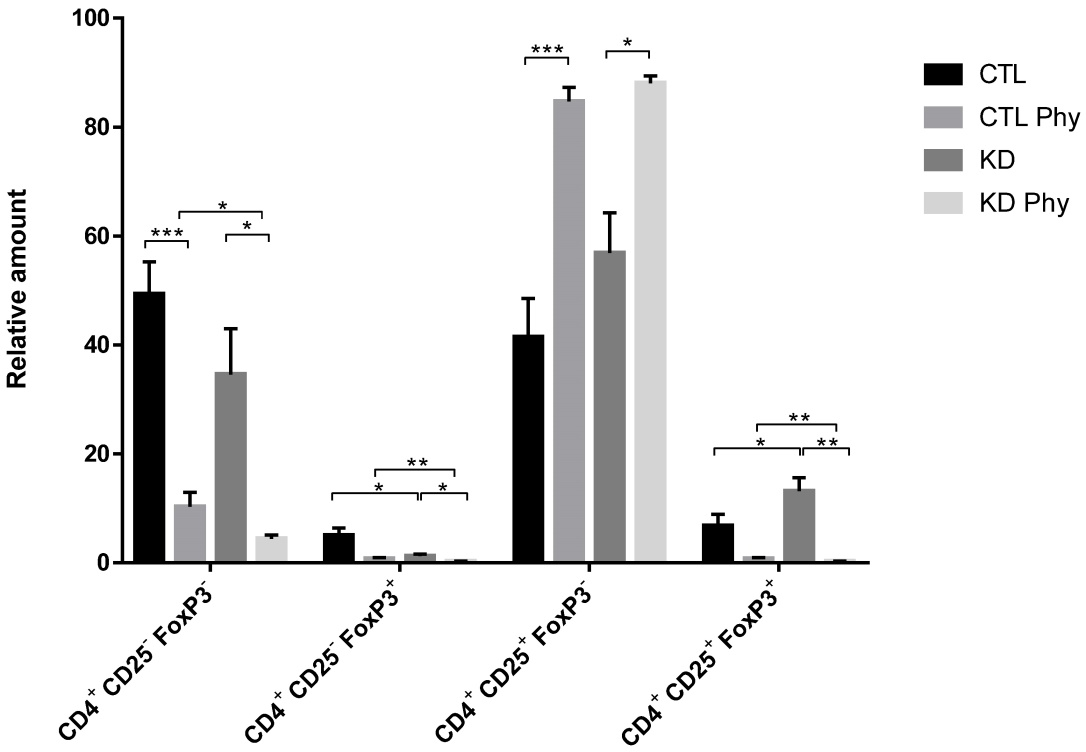
** MacroH2A1 KD conditioned media trigger CD4^+^/CD25^+^/FoxP3^+^ Treg cells expansion.** A. T cells isolated from peripheral blood mononuclear cells of healthy volunteers were exposed to the culture media taken from CTL cells, macroH2A1 KD cells or CTL cells exposed to the conditioned media (CM) of KD cells. All cell lines were pretreated, or not, with PPP inhibitor Physcion (Phy, 5 μM, 48h). The T cells were activated and stained with CD4^+^, CD25^+^ and FoxP3^+^ specific antibodies before flow cytometry. Results are expressed as the percentage (%) of the parental population. N = 8; p < 0.05, ** p < 0.01. *** p < 0.001, comparisons as indicated.

Tregs mediate their suppressive function not only through cell-to-cell contact, but also via soluble mediators that provide a positive feedback loop for immune suppressive mechanisms by generating inducible Tregs cells from CD4^+^/CD25^-^T effector cells 43. Therefore, we investigated the potential of macroH2A1 KD CSCs to activate CD4^+^/CD25^+^/FoxP3^+^ Tregs. We isolated fresh T cells from healthy donor blood and incubated them for 72 h with either CTL media or KD media. Using the flow cytometry gating strategy depicted in Figure S10, we observed a significant increase in the frequency of activated CD4^+^/CD25^+/^FoxP3^+^ Tregs upon treatment with KD media compared to control media (Figure 8). This increase was paralleled by a decrease in CD4^+^/CD25^-/^FoxP3^+^ T cells, indicating that conditioned media form KD cells triggers Treg generation from these precursors in the T cell pool. Altogether these data suggest that Tregs are activated in a paracrine manner by macroH2A1 KD HCC cells. Interestingly, pre-treatment of Huh-7 CD and macroH2A1 KD cells with PPP inhibitor Physcion (5 μM, 48h) generated a conditioned medium that nearly completely suppressed the frequency of activated CD4^+^/CD25^+/^FoxP3^+^ Tregs, while massively increasing in parallel the number of CD4^+^/CD25^+^/FoxP3^-^ T cells (Figure 8). Physcion also significantly inhibited the formation of the pools of CD4^+^/CD25^-/^FoxP3^-^ and CD4^+^/CD25^-/^FoxP3^+^ T cells (Figure 8). The functionality of CD4^+^/CD25^+/^FoxP3^+^ Tregs was also analyzed in carboxyfluorescein succinimidyl ester (CFSE)-based suppression assays. Concurrent with co-incubation with CTL or KD media, CFSE-labeled CD4^+^/CD25^-^ T responder cells were mixed with CD4^+^/CD25^+/^FoxP3^+^ Tregs in presence of Treg suppression inspector (anti-Biotin MACSiBead™ particles loaded with biotinylated CD2, CD3, and CD28 antibodies), and then proliferation rate of responder cells was measured. We did not detect differences in the suppressive activity of Tregs from the CTL or the KD group (Figure S11). In summary, media produced from HCC cells KD for macroH2A1 harboring lead to an expansion in the number of activated Tregs, without alterations in their suppressive function.

### HCC harboring low macroH2A1 expression displays decreased cellular senescence

MacroH2A1 modulates the transcriptional activity of IL-6 and IL-8 38, and these cytokines are important effectors of the senescence associated secretory phenotype (SASP) 7. SASP renders cells vulnerable to immune-mediated clearance 7. Therefore, we sought to determine if altered macroH2A1 isoform (macroH2A1.1 and macroH2A1.2) levels in HCC patient tissue samples correlate with altered frequency of tumor resident senescent cells. We found that a significantly higher number of cells immunopositive for β-galactosidase (β-gal), a key marker of cellular senescence, paralleled the progression of liver disease (control < cirrhosis < HCC) and the increased immunopositivity for macroH2A1 isoforms in a cohort of patients bearing differentiated/encapsulated tumors that we previously described 23, 24 (Figure S8). Moreover, p16 has been identified as main player in the cellular senescence response in the liver cellular senescence program, which in turn is involved in tumour immune surveillance 7, 8. Here we found that p16 was expressed at lower levels in the previously examined liver biopsies of patients with poorly differentiated HCC and low macroH2A1 expression compared to those with well differentiated HCC and high macroH2A1 expression 26 (Figure 7, Figure S9). Altogether, these immunohistochemistry data suggest that macroH2A1 expression positively correlated with cellular senescence in human liver. Moreover, cellular senescence phenotype correlates positively with HCC differentiation.

## Discussion

A high presence of CSCs in the liver of patients with HCC is associated with HCC relapse and metastasis 2. Here, we show for the first time that liver CSC-like cells can reprogram neighboring HCC cells into other CSC-like cells that can evade the adaptive immune system via a combination of epigenetic (macroH2A1) and paracrine mechanisms. MacroH2A1 is a marker of well-differentiated HCC and of non-tumor/tumor liver tissue senescence 23, 24; however, our previous and current data suggest that macroH2A1 expression is dramatically decreased or absent in a wide range of independent poorly-differentiated human HCCs 16, although the correlation between macroH2A1 immunostaining and HCC encapsulation that we found needs validation in a larger cohort. What triggers macroH2A1 down-regulation and subsequent macroH2A1-dependent transformation of HCC cells into CSC-like cells remains unknown. Previous evidence suggest that loss of macroH2A1 might promote a relaxed chromatin state that is poised for chromatin remodeling to enhance accessibility to pro-inflammatory transcription factors (NF-κB), and re-expression of reprogramming genes that together might lead to a CSC-like phenotype 17, 45, 46.

The tumor secretome consists of diffusible factors derived from CSCs, cancer cells and the surrounding stroma that determine cancer progression. Proteomic analysis of pancreatic CSCs previously revealed 72 differentially regulated proteins compared to the parental cells 47. To the best of our knowledge, the liver CSC secretome has not been studied. Our analysis of a panel of 507 human cytokines, chemokines, adipokine, growth factors and other soluble molecules in Huh-7 HCC cells with macroH2A1 KD revealed a significant down-regulation of 92 out of 94 differentially expressed molecules (98%), including IL-6 and IL-8. The expression levels of these interleukins are under the specific control of macroH2A1.1 isoform - and not macroH2A1.2 - 38; we found macroH2A1.1 expressed at higher levels in Huh-7 compared to HepG2 cells. HepG2 cells do not produce IL-6 (Figure S6, 34). Interestingly, secretomic effects were not observed in HepG2 cells KD for macroH2A1. Also, macroH2A1 KD HepG2 cells, unlike Huh-7 cells, were unable to transfer chemoresistance paracrinally, which might be consistent with lower levels of macroH2A1.1 38 in this cell line. HepG2 express a wild type tumor suppressor p53, while Huh-7 cells express a mutant dominant negative p53 (Y220C) 48. p53 mutations are frequent in human HCC and are associated to a shorter survival rate compared to the one of wild type p53 carriers 49; they are also major components in the establishment of CSC entity 50. HepG2 and Huh-7 cells KD for macroH2A1 present also metabolic differences: while both cell lines display increased glycolytic pathways, a more significant upregulation of pentose phosphate pathway (PPP) with an increased abundance of redox electron carriers (NADP, NADPH) is specifically observed in Huh-7 (15, this study), while an increased intracellular acetyl-CoA and lipid accumulation is specifically observed in HepG2 (15, this study). p53 is a strong inhibitor of PPP: p53 protein binds to glucose-6-phosphate dehydrogenase (G6PD), the first and rate-limiting enzyme of the PPP, and prevents the formation of the active dimer 51. Cancer-associated p53 mutants lack the G6PD-inhibitory activity 51. Pre-malignant senescent hepatocytes produce SASP that renders these cells vulnerable to immune-mediated clearance 7. Our working hypothesis, supported by the data on PPP-dependent chemokine-cytokine production, is that CSC-like macroH2A1 KD Huh-7 cells, which are p53 mutant and thus display an over-activated PPP, are able to produce an “anti-SASP” - depleted in IL-6, IL-8 and other effectors, triggering a potential mechanism of immune evasion. Consistent with our data, macroH2A1-depleted IMR90 cells do not senesce in response to SASP factor-containing conditioned media from senescent cells, and do not activate SASP genes 38. It is also possible that over-activated PPP and deficient macroH2A1 expression trigger positive epistatic effects on the secretome of HCC cells.

How macroH2A1 KD induces general inhibition of secreted factors in HCC cells? Surprisingly, integrated transcriptomic and secretomic analysis uncovered a low degree of significant concordance between mRNA and protein expression levels, ~11% (10 out of 94 secreted factors). Cytokine expression and production is typically regulated by extensive post-transcriptional regulation 52, 53. Moreover, cellular perturbations such as endoplasmic reticulum stress, oxidative stress and DNA damage coordinately regulate macroH2A1-dependent production of IL-6 and IL-8 upon K-Ras-dependent oncogenic transformation of fibroblasts 38. The elucidation of the transcription/post-transcriptional mechanisms leading to the observed cytokine/chemokine depletion in macroH2A1 KD-induced CSC-like cells warrants further studies. Regardless, the cytokine-depleted secretome is related to immune evasion. While elevated levels of cytokines produced by tumor cells enhance CSC proliferation and the differentiation 39, our data suggest the possibility that decreased levels of cytokines produced by CSC-like cells might favor quiescence, pluripotency and chemoresistance - properties that are transcriptionally programmed into neighboring cancer cells. If this holds true, interventions for general neutralizations of chemokines/cytokines within the tumor parenchyma might be ineffective on the CSC extracellular niche.

Given the rarity of CSCs within the HCC mass 3, it will prove challenging to obtain spatially resolved data about the gradients of cytokine and chemokine levels *in vivo*. Technological advancements to profile low-molecular-mass proteins are required for an unbiased full characterization of the CSC secretome. Our findings provide evidence that the macroH2A1-dependent CSC-like secretome depleted in severable immune mediators leads to CD4^+^/CD25^+^/FoxP3^+^ Treg expansion, without changes in their suppressive activity, and simultaneous curtailment of CD4^+^CD25^-^/FoxP3^+^ T cells in human T lymphocyte pools. Treg development is classically dependent on IL-2 54. IL-2 levels were unchanged in macroH2A1 KD CSC-like cells (*data not shown*). Noteworthy, the only two secreted factors augmented in the conditioned medium from CSC-like cells were GDF15 and lipocalin-2. Both factors can promote CD4^+^/CD25^+^/FoxP3^+^ Treg activation, CSC-like properties and immune escape 55-59. Our data thus suggest the existence of a complex scenario where the macroH2A1 KD-induced CSC-like cells inhibit T cell maturation through downregulation of secreted immune mediators (Figure 6A), in concert with CD4^+^/CD25^+^/FoxP3^+^ Treg expansion due to the increased secreted levels of GDF15 and/or lipocalin-2.

We provide also data that blocking the hyperactivation of PPP in macroH2A1 KD Huh-7 cells using PPP specific inhibitor Physcion led to restoration of downregulated cytokines (IL-6, IL-8) and nearly completely suppressed CD4^+^/CD25^+/^FoxP3^+^ Tregs, suggesting a previously unappreciated link between energy metabolism and chemokine/cytokine production in HCC cells.

During tissue injury, antigen‐specific CD4^+^/CD25^-^ T cells participate in the control of tissue homeostasis in concert with CD8^+^ T cells, macrophages, and NK cells at the inflammatory site. Genetic experiments have revealed that functional senescence surveillance is dependent on an intact adaptive immune response. In CD4^+^ knockout mice, the immune clearance of premalignant senescent hepatocytes is abrogated, favoring HCC development 7. We propose that the observed decreased recruitment of CD4^+^ T cells in macroH2A1-deficient HCC foci contributes, at least in part, to adaptive immune system evasion. A limitation of our study is that it does not address the complex mixture of non-transformed cell types and the extracellular matrix of the TME, as we focus only on major HCC-CSC and CSCs-T lymphocyte cell-cell crosstalk. Further work in 3D tissue models, which might allow microvasculature development and quantitative assessment of cell-cell and cell-drug interactions in a physiologically realistic TME, is required to confirm these findings. Approaches aiming at targeting cellular metabolism in conjunction with chemotherapeutic drugs 60, or ablating CD44 expression to inhibit glycolysis and the PPP 61, or modulating T cells activity through optogenetics 62, are innovative strategies that might be useful to treat macroH2A1-negative aggressive HCCs.

## Abbreviations

HCC: hepatocellular carcinoma
CSC: cancer stem cells
Treg: regulatory T cells
KD: knockdown
CCM: central carbon metabolism
PPP: pentose phosphate pathway
