# Supplementary material for: Loss of histone macroH2A1 in hepatocellular carcinoma cells promotes paracrine-mediated chemoresistance and CD4+CD25+FoxP3+ regulatory T cells activation
Source: Theranostics. 2020 Jan 1;10(2):910–24. doi: 10.7150/thno.35045 (PMC6929991; doi:10.7150/thno.35045)
Supplement: Supplementary file 1 — Supplementary figures and tables. [file thnov10p0910s1.pdf]

Supplemental Material

Loss of histone macroH2A1 in hepatocellular carcinoma cells promotes paracrine-mediated chemoresistance and $CD4^{+}CD25^{+}FoxP3^{+}$ regulatory T cells activation

Oriana Lo Re[1,2*], Tommaso Mazza[3*], Sebastiano Giallongo[1,2], Paola Sanna[1], Francesca Rappa[4], Tu Vinh Luong[5], Giovanni Li Volti[6], Adela Drovakova[7,8], Tania Roskams[9], Matthias Van Haele[9], Emmanuel Tsochatzis[10], Manlio Vinciguerra[1,10$]

[1] International Clinical Research Center, St' Anne's University Hospital, Brno, Czech Republic;

[2] Department of Biology, Faculty of Medicine, Masaryk University, Brno, Czech Republic;

[3] IRCCS Casa Sollievo della Sofferenza, Laboratory of Bioinformatics, San Giovanni Rotondo (FG), Italy;

[4] Department of Experimental Biomedicine and Clinical Neurosciences, University of Palermo, Palermo, Italy;

[5] Department of Histopathology, Royal Free London NHS Foundation Trust, London, United Kingdom;

[6] Department of Biomedical and Biotechnological Sciences, University of Catania, Catania, Italy;

[7] Institute of Biophysics, Academy of Sciences of the Czech Republic, Brno, Czech Republic;

[8] Department of Animal Physiology and Immunology, Institute of Experimental Biology, Masaryk University, Brno, Czech Republic;

[9] Translational Cell & Tissue Research Unit, Department of Imaging & Pathology, Katholieke Universiteit Leuven, Leuven, Belgium;

[10] UCL Institute for Liver and Digestive Health, Royal Free Hospital, London, United Kingdom.

Supplementary Figures

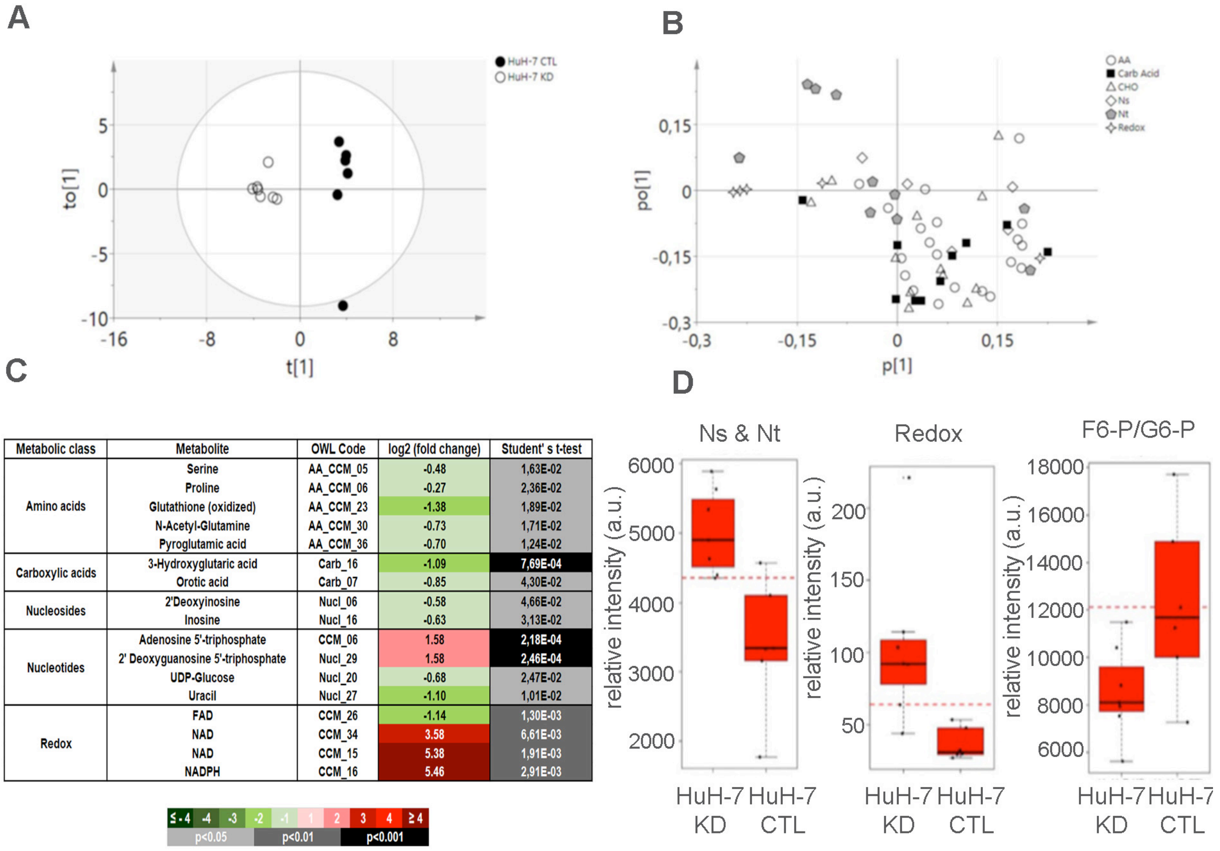

Metabolic class	Metabolite	OWL Code	log2 (fold change)	Student' s t-test
Amino acids	Serine	AA_CCM_05	-0.48	1,63E-02
	Proline	AA_CCM_06	-0.27	2,36E-02
	Glutathione (oxidized)	AA_CCM_23	-1.38	1,89E-02
	N-Acetyl-Glutamine	AA_CCM_30	-0.73	1,71E-02
	Pyroglutamic acid	AA_CCM_36	-0.70	1,24E-02
Carboxylic acids	3-Hydroxyglutaric acid	Carb_16	-1.09	7,69E-04
	Orotic acid	Carb_07	-0.85	4,30E-02
Nucleosides	2'Deoxyinosine	Nucl_06	-0.58	4,66E-02
	Inosine	Nucl_16	-0.63	3,13E-02
Nucleotides	Adenosine 5'-triphosphate	CCM_06	1.58	2,18E-04
	2' Deoxyguanosine 5'-triphosphate	Nucl_29	1.58	2,46E-04
	UDP-Glucose	Nucl_20	-0.68	2,47E-02
	Uracil	Nucl_27	-1.10	1,01E-02
Redox	FAD	CCM_26	-1.14	1,30E-03
	NAD	CCM_34	3.58	6,61E-03
	NAD	CCM_15	5.38	1,91E-03
	NADPH	CCM_16	5.46	2,91E-03

Figure S1. Metabolomic analysis of one carbon metabolism in HCC cells. A. Score scatter plot and B. Loading scatter plot of the OPLS-DA model of Huh-7 control (CTL) versus macroH2A1 KD cells. C. Significantly altered metabolites in Huh-7 KD compared to Huh-7 control CTL cells. FAD (Flavin adenine dinucleotide), NAD (nicotinamide adenine dinucleotide), NADP (NAD phosphate), NADPH (NADP reduced). D. Boxplots of nucleosides and nucleotides (Ns & Nt), redox electron carriers (Redox), and the ratio Fructose-6-phosphate/Glucose-6-phosphate in Huh-7 KD versus Huh-7 CTL cells.

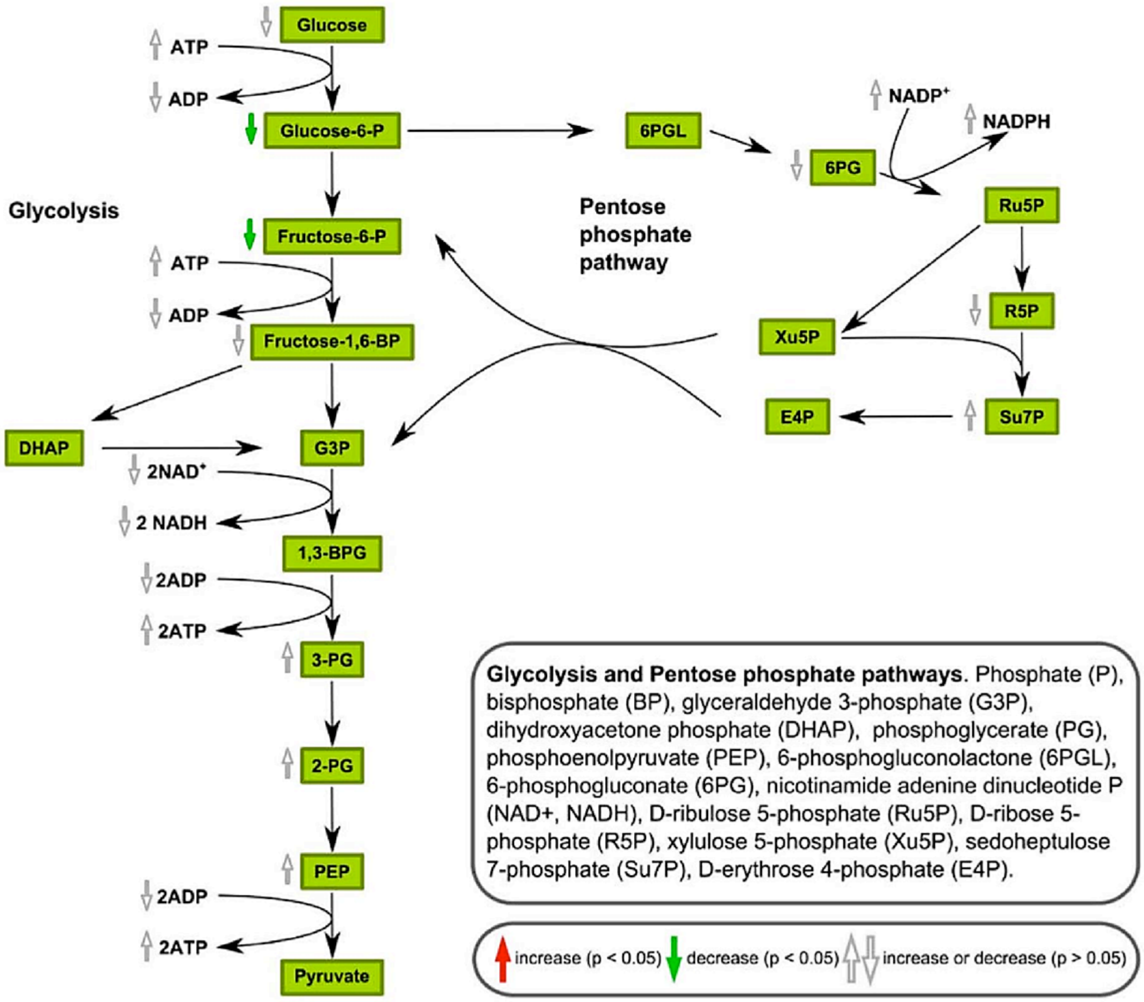

Figure S2. Changes in glycolysis and the pentose phosphate pathway (PPP) in Huh-7 macroH2A1 knockdown (KD) compared to control (CTL) cells. Red arrows indicate significant increases ($P < 0.05$), green arrows indicate significant decreases ($P < 0.05$), and white arrows indicate non-significant changes ($P > 0.05$).

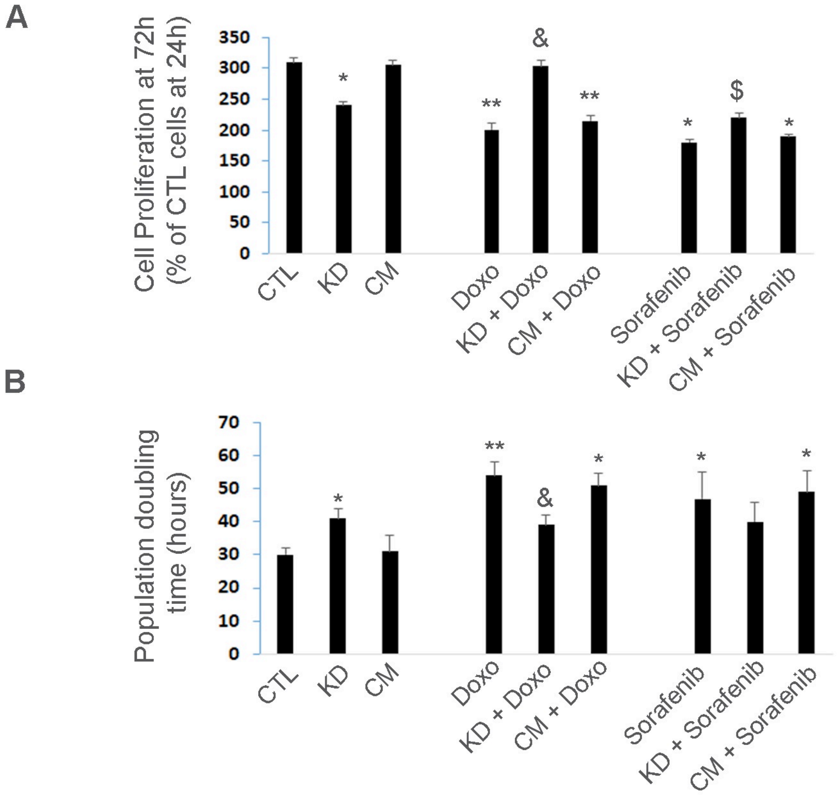

Figure S3. HepG2 macroH2A1 KD cells do not confer chemoresistance to parental cells. A. Three experimental conditions: control (CTL), KD and CTL cells plus KD conditioned medium (CM). C. MTT assay in CTL, KD or CTL + CM cells incubated with or without vehicle (DMSO), 2 µM Doxorubicin (Doxo) or 1 µM Sorafenib for 72 h. Data represent the mean cell proliferation ± s.d. relative to CTL cells at 24 h. N=3. D. Population doubling time in CTL, KD or CTL + CM cells incubated with or without vehicle (DMSO), 2 µM Doxorubicin (Doxo) or 1 µM Sorafenib for 72 h. Data represent the mean cell proliferation ± s.d. N = 3. *P < 0.05, ** P < 0.01 relative to CTL; $^{\&}$ P < 0.05 relative to Doxo; $^{\$}$ P < 0.05 relative to Sorafenib.

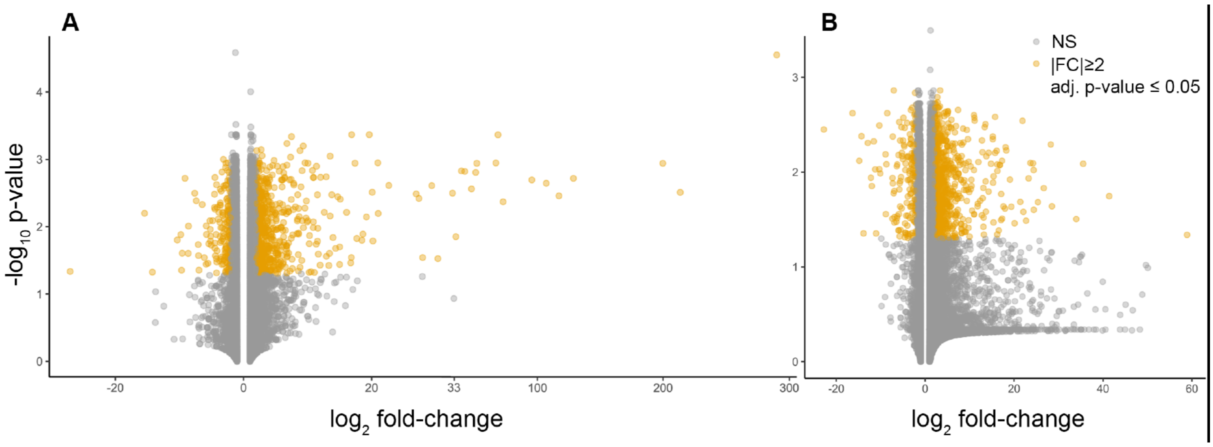

Figure S4. Volcano plot representing differentially expressed genes between macroH2A1 KD versus control cells (A), and between conditioned media (CM) KD treated cells versus control Huh-7 cells. Orange dots: significant and differentially expressed genes. Grey dots: statistically nonsignificant (NS) expressed genes. 783 and 987 genes were significantly and differentially expressed (|FC|≥2, adj. p-value ≤ 0.05) over a total number of 26439 screened genes, in macroH2A1 KD or CM KD versus control Huh-7 cells, respectively.

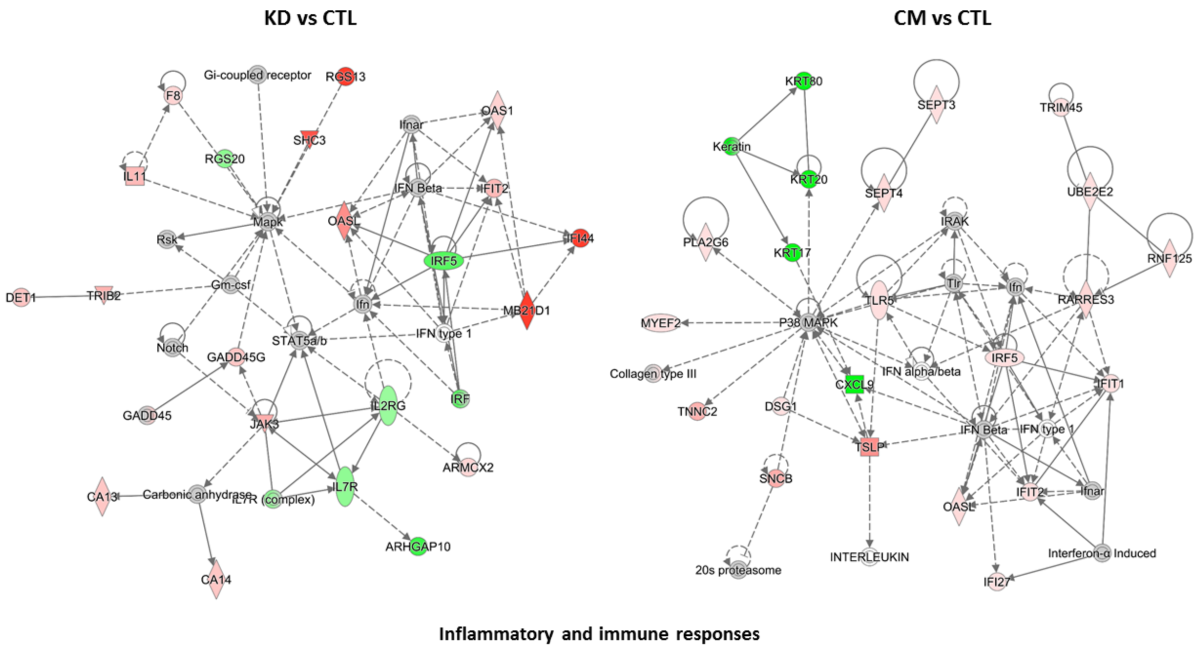

Figure S5. Functional enrichment analysis of commonly and differentially expressed genes in control (CTL), macroH2A1 knockdown (KD) and conditioned media (CM) Huh-7 cells. C. Networks of differentially expressed genes in KD vs CTL (left) and CM vs CTL (right) involved in the Inflammatory and Immune Responses functions. Over-expressed and down-regulated molecules are colored in red and green, respectively.

A

POS	POS	NEG	NEG	ENA-78	GCSF	GM-CSF	GRO	GRO-α	I-309	IL-1α	IL-1β
POS	POS	NEG	NEG	ENA-78	GCSF	GM-CSF	GRO	GRO-α	I-309	IL-1α	IL-1β
IL-2	IL-3	IL-4	IL-5	IL-6	IL-7	IL-8	IL-10	IL-12 P40/p70	IL-13	IL-15	INF-Y
IL-2	IL-3	IL-4	IL-5	IL-6	IL-7	IL-8	IL-10	IL-12 P40/p70	IL-13	IL-15	INF-Y
MPC-1	MCP-2	MPC-3	MCSF	MDC	MIG	MIP-1δ	RANTES	SCF	SDF-1	TARC	TGF-β1
MPC-1	MCP-2	MPC-3	MCSF	MDC	MIG	MIP-1δ	RANTES	SCF	SDF-1	TARC	TGF-β1
TNFα	TNF-β	EGF	IGF-I	Angiogenin	Oncostatin M	Thrombopoietin	VEGF	PDGF BB	Leptin	NEG	POS
TNF-α	TNF-β	EGF	IGF-I	Angiogenin	Oncostatin M	Thrombopoietin	VEGF	PDGF BB	Leptin	NEG	POS

B

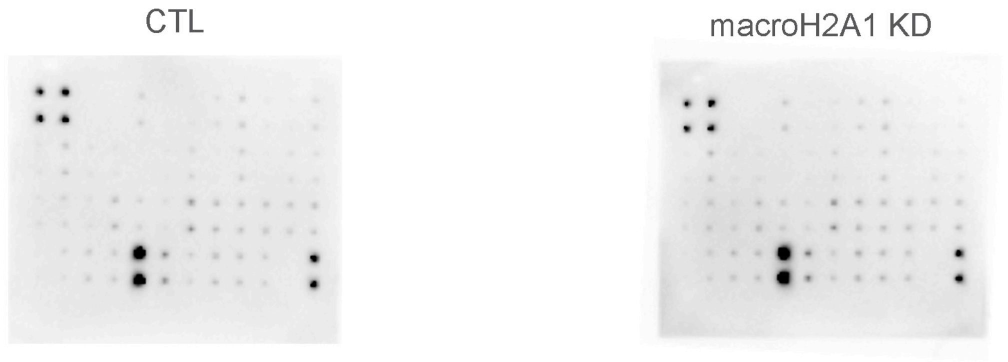

Figure S6. Conditioned media (CM) from HepG2 macroH2A1 KD cells has similar cytokine/chemokine content to HepG2 control cells. A. 48 human cytokines/chemokines were analyzed in the Huh-7 control (CTL) and KD cell supernatants.

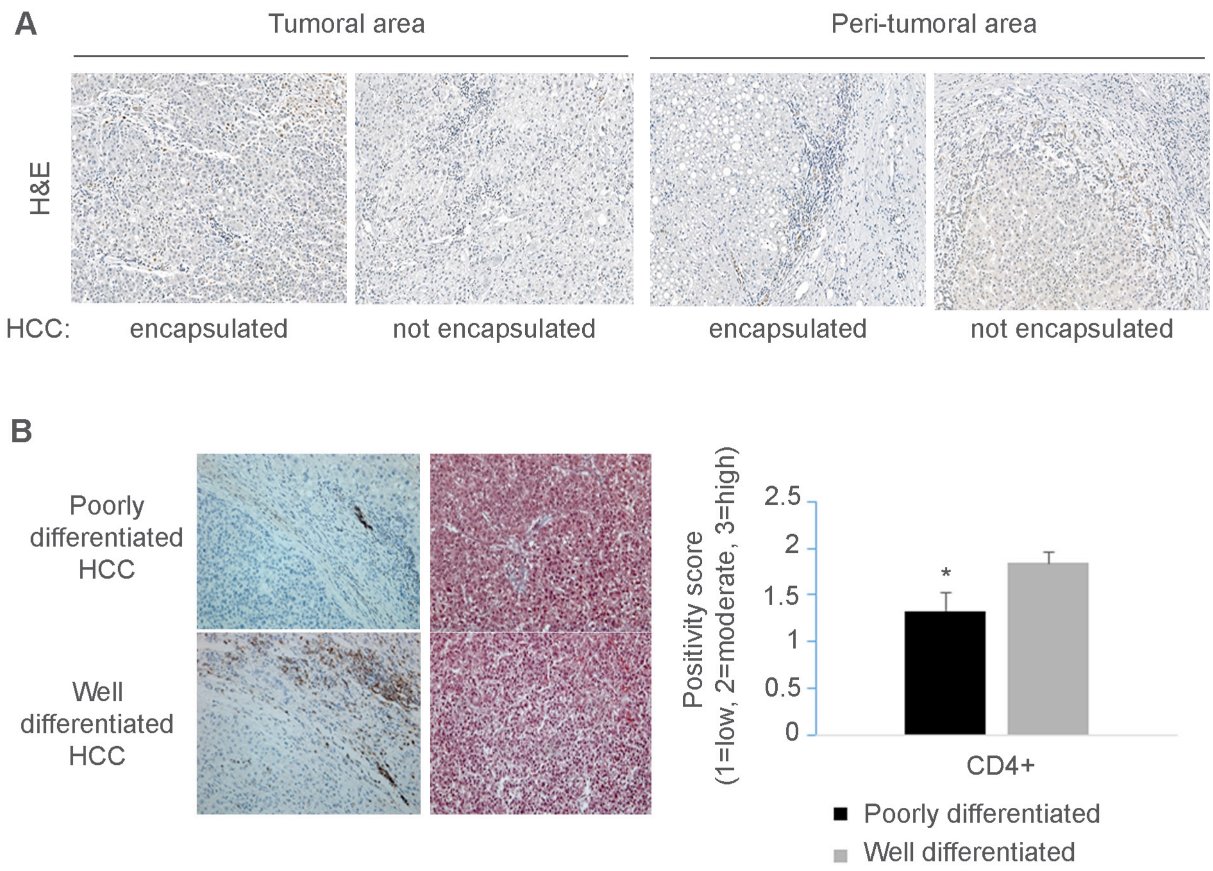

Figure S7. HCC harboring low macroH2A1 expression displays decreased CD4(+) lymphocyte infiltration. A. Representative hematoxylin & eosin (H&E) staining of encapsulated versus non-encapsulated Hepatocellular carcinoma (HCC) tumors from explanted livers of patients undergoing liver transplantation as in Figure 2. Magnification, 100 X. B. Immunohistochemical staining for $CD4^{+}$ cells and H&E staining on poorly-differentiated (n=18) and well-differentiated (n=16) HCC samples, from a previously characterized cohort [1]. The right panel shows a corresponding semi-quantitative evaluation of positivity scores (1=low, 2=moderate, 3=high) for CD4 staining for the same cases. *$p < 0.05$ compared to well differentiated.

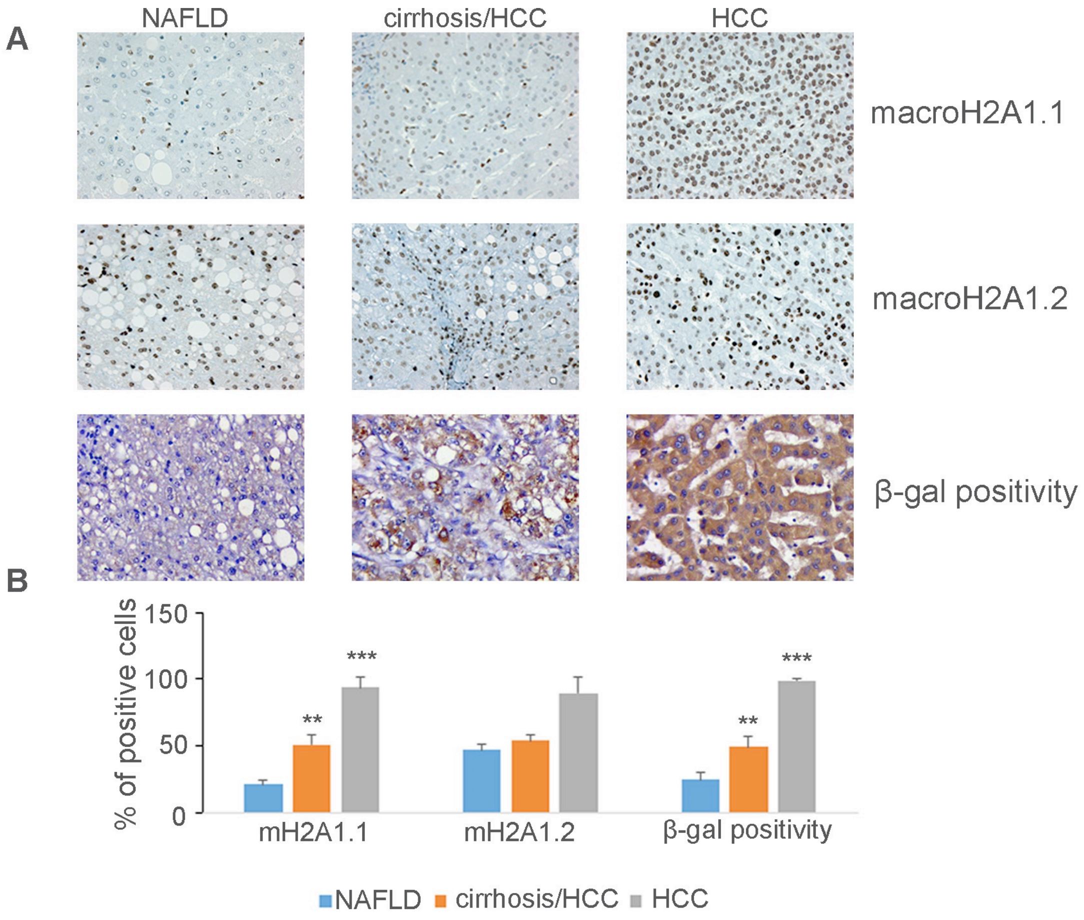

Figure S8. A. Representative pictures of macroH2A1.1, macroH2A1.2 and β-gal immunostaining of human liver samples with NAFLD, cirrhosis, and HCC (n = 10 per condition) [2, 3]. All HCC cells were highly positive for macroH2A1.1, macroH2A1.2 and β-gal; positivity of hepatocytes with NAFLD was significantly lower and was intermediate in viral cirrhosis. Magnification: 400 X. B. After performing quantitative analysis, the results were expressed in a semiquantitative scale (0, 0%; 1, 1%–33%; 2, 34%–66%; 3, 67%–100%). Data were expressed as means ± SE. **, $P < 0.01$ and ***, $P < 0.001$.

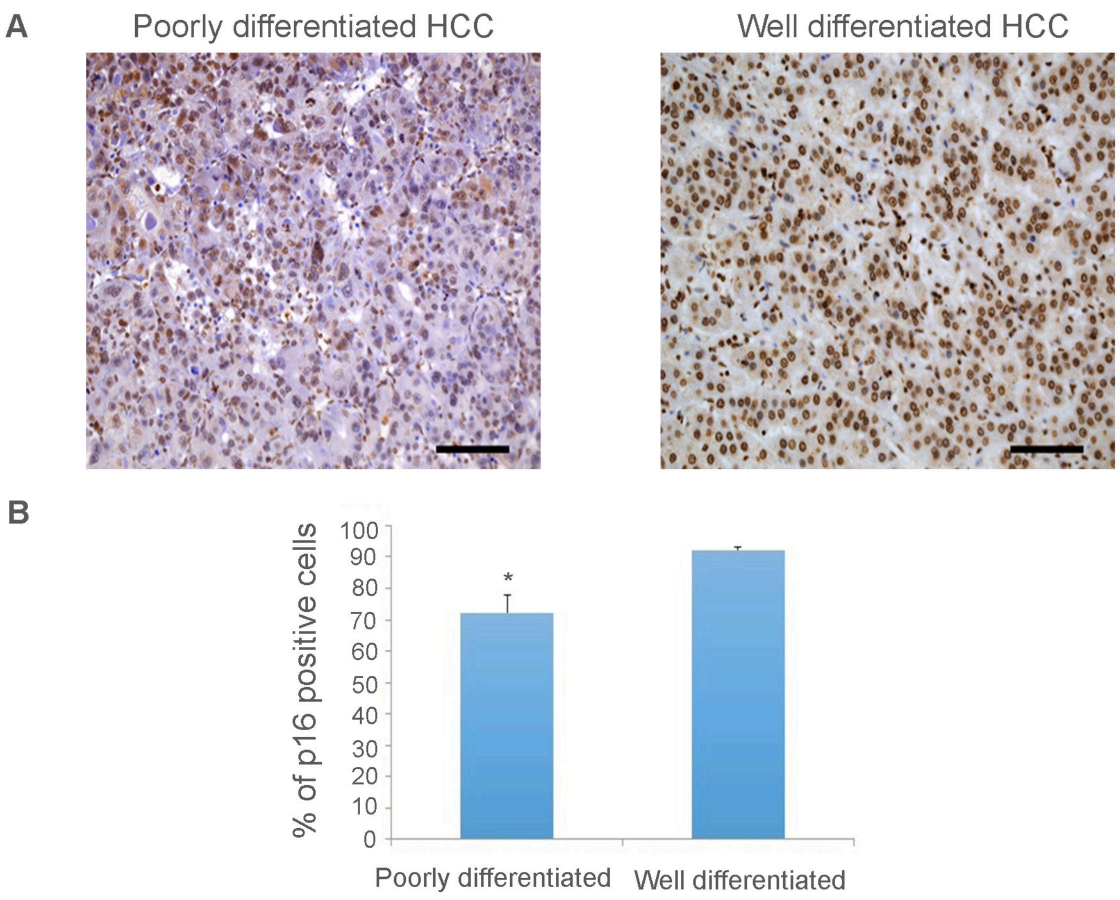

Figure S9. A. Representative immunohistochemical staining for p16 cells on poorly differentiated (n = 18) and well-differentiated (n = 16) HCC samples, from a previously characterized cohort [1]. After performing quantitative analysis, the results were expressed in a semiquantitative scale (0, 0%; 1, 1%–33%; 2, 34%–66%; 3, 67%–100%). Data were expressed as means ± SE. *p<0.05 compared to well differentiated.

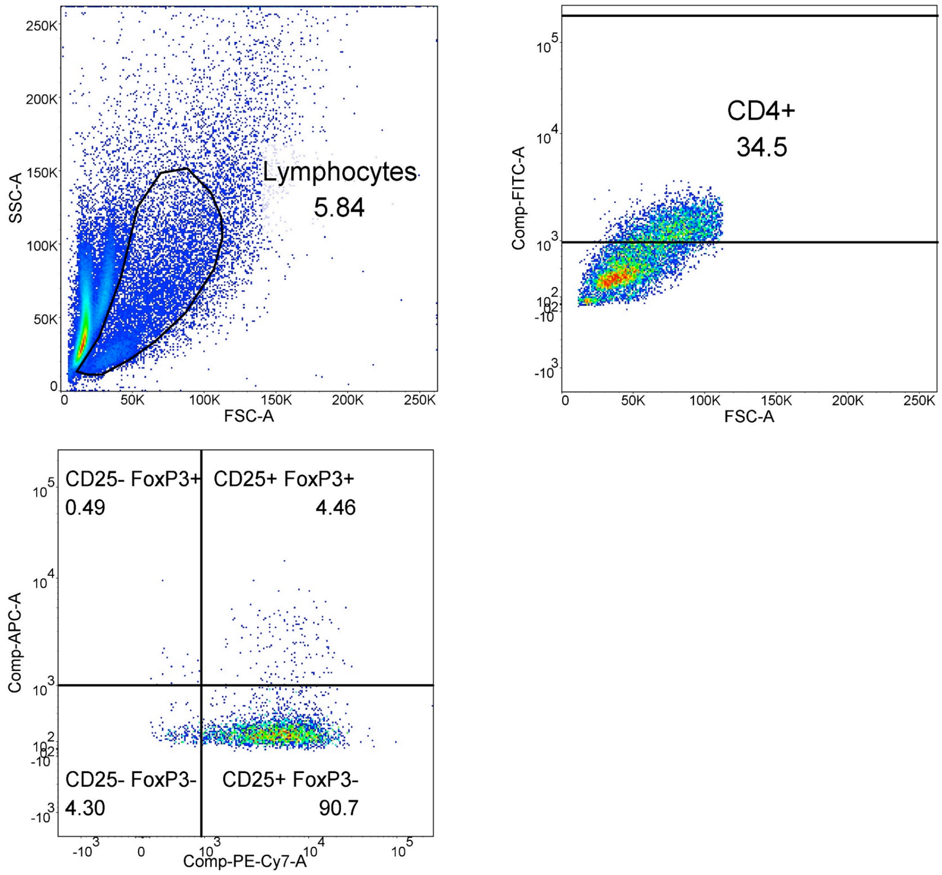

Figure S10. MacroH2A1 KD conditioned media trigger $CD4^+/CD25^+/FoxP3^+$ Treg cells expansion. T cells isolated from peripheral blood mononuclear cells of healthy volunteers were exposed to the culture media as described in Figure 8 Legend. The Figure shows a representative flow cytometric plot. Cells were stained for Treg markers with antibody combination CD4/CD25/FoxP3 and gated for lymphocytes (SSC-A, FSC-A) (upper left panel) and for $CD4^+$ (upper right panel). Four populations were then gated: $CD4^+/CD25^{-/}FoxP3^-$, $CD4^+/CD25^{-/}FoxP3^{+,}$ $CD4^+/CD25^+/FoxP3^-$ and $CD4^+/CD25^{+/}FoxP3^+$ cells (lower left panel).

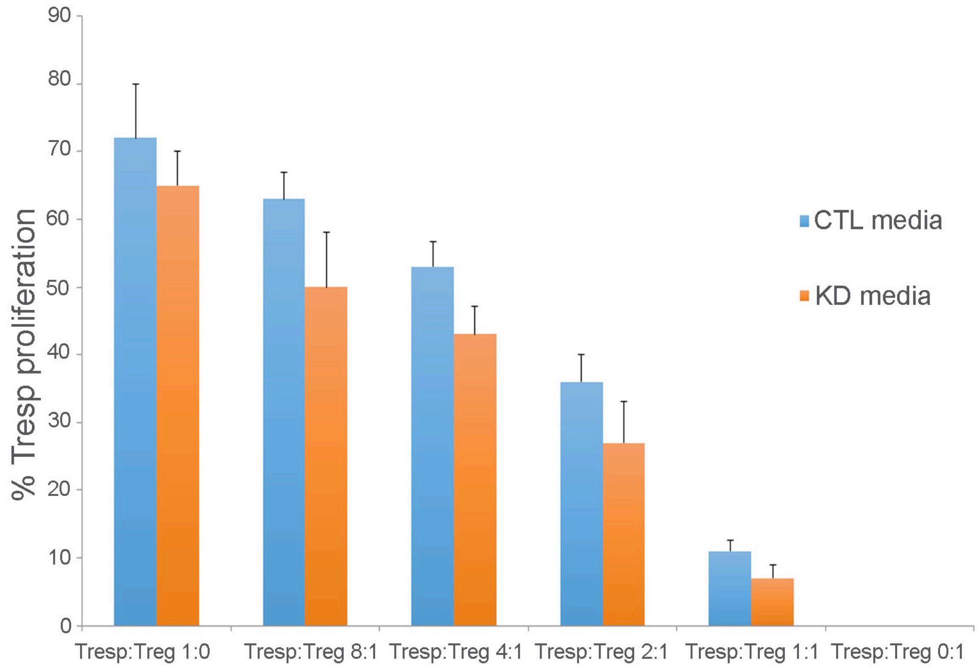

Figure S11. CTL and macroH2A1 KD conditioned media trigger similar CD4$^+$/CD25$^+$/FoxP3$^+$ Treg cells functional activation. The suppressing function was analyzed by CFSE-labeled CD4+ CD25− T cells co-cultured with CD4+ CD25+ Tregs and Treg Suppression Inspector, which was composed of anti-biotin MACSiBead™ particles preloaded with biotinylated anti-CD2, anti-CD3, and anti-CD28 antibodies. After 4 days of culture, proliferation was measured based on CFSE signal.

Supplemental Tables

Supplemental Table 1. Fold changes and unpaired Student's t-test of each individual metabolite and of each metabolic class.

Class	Metabolite	Fold change	Log_2 (fold change)	Student's t-test (p)
AA	Serine	1.06	0.09	6.78E-01
	Proline	1.05	0.07	6.13E-01
	**Valine**	**1.29**	**0.36**	**4.87E-02**
	Threonine	0.93	-0.11	6.65E-01
	Taurine	0.68	-0.56	1.61E-01
	Isoleucine	0.99	-0.02	9.47E-01
	Leucine	0.94	-0.09	6.71E-01
	Asparagine	0.96	-0.05	8.48E-01
	Glutamine	0.22	-2.20	2.69E-01
	Methionine	0.87	-0.20	4.51E-01
	Phenylalanine	1.28	0.36	9.13E-02
	Tyrosine	1.00	0.00	9.97E-01
	Tryptophan	1.14	0.19	4.29E-01
	Glutathione (reduced)	1.82	0.87	1.97E-01
	Glutathione (oxidized)	0.10	-3.36	2.61E-01
	S-Adenosylhomocysteine	0.93	-0.10	9.51E-01
	N-Acetylglutamic acid	1.44	0.53	2.61E-01
	N-Acetyl-glutamine	0.37	-1.44	2.25E-01
	Pyroglutamic acid	0.78	-0.36	2.70E-01
	Hippuric acid	1.38	0.47	1.96E-01
Carbohydrates	2,3-bisphosphoglycerate	2.00	1.00	4.75E-01
	2-phosphoglycerate / 3-phosphoglycerate	1.11	0.15	8.46E-01
	6-Phosphogluconate	0.07	-3.86	2.70E-01
	**D-Mannose 6-phosphate and 1D-myo-Inositol 3-phosphate**	**0.16**	**-2.61**	**3.02E-02**
	D-Ribose 5-phosphate	0.12	-3.12	9.58E-02
	Fructose 1,6-bisphosphate	0.40	-1.33	3.85E-01
	**Fructose 6-phosphate**	**0.03**	**-5.28**	**1.80E-02**
	Glucose	0.68	-0.56	5.66E-02
	**Glucose-6-phosphate**	**0.20**	**-2.30**	**3.83E-02**
	Phosphoribosyl pyrophosphate	0.18	-2.44	1.80E-01
	Sedoheptulose	1.33	0.41	3.78E-01
	Sorbitol	0.79	-0.34	3.99E-01
Carboxylic acids	3-Hydroxyglutaric acid	1.00	0.01	9.81E-01
	**4-Pyridoxic acid**	**1.54**	**0.63**	**4.29E-02**
	5-Methyltetrahydrofolic acid	1.32	0.40	3.78E-01
	Citrate / iso-Citrate	0.92	-0.13	6.66E-01
	Fumaric acid	0.87	-0.20	1.13E-01
	Malate	0.86	-0.22	6.59E-02
	N-Acetylneuraminic acid	0.93	-0.10	7.96E-01
	Orotic acid	1.19	0.25	4.89E-01
	Phosphoenolpyruvate	1.94	0.95	4.51E-01
	Succinate	0.58	-0.79	6.79E-02
Fatty acid ester	**Acetyl-coenzyme A**	**25.77**	**4.69**	**3.15E-02**
Nucleosides	2'Deoxyinosine	0.79	-0.34	4.58E-01
	Guanosine	0.29	-1.81	5.63E-02
	Inosine	0.58	-0.79	1.24E-01
	Uridine	0.99	-0.02	9.69E-01
	Xanthosine	0.60	-0.73	4.24E-01
Nucleotides	Adenosine 5'-diphosphate	0.61	-0.71	5.34E-01
	ADP-Glucose	1.21	0.28	2.49E-01
	Adenosine 5'-monophosphate	0.50	-0.99	5.36E-01
	Adenosine 5'-triphosphate	1.29	0.36	2.65E-01
	**Cytidine 5'-triphosphate**	**1.53**	**0.61**	**4.26E-02**
	2'-Deoxyguanosine 5'-triphosphate	1.28	0.36	2.83E-01
	Guanosine 5'-diphosphate	0.66	-0.60	5.27E-01
	Guanosine 5'-triphosphate	1.29	0.37	1.99E-01
	UDP-Glucose	0.93	-0.11	7.21E-01
	Uracil	0.87	-0.20	2.99E-01
	Uridine 5'-triphosphate	1.26	0.33	3.13E-01
Redox electron carriers	Flavin adenine dinucleotide	0.70	-0.51	1.67E-01
	Nicotinamide adenine dinucleotide	0.91	-0.14	2.36E-01
	Nicotinamide adenine dinucleotide reduced	0.28	-1.82	2.71E-01
	Nicotinamide adenine dinucleotide phosphate	1.35	0.44	1.31E-01
	Nicotinamide adenine dinucleotide phosphate reduced	1.44	0.53	1.18E-01

Supplemental Table 2. Clinic-pathological features of 20 patients (N=10 with encapsulated HCC + N=10 with not encapsulated/infiltrative HCC) undergoing liver transplantation for HCC in the period 1995-2000 at the Royal Free Hospital (London, UK), included in this study. Demographic information and HCC etiology is shown for encapsulated versus non-

encapsulated cases. Viral hepatitis: HBC, HCV, HBV/HCV, HBC/HDV. Cryptogenic: of unknown origin; Alpha 1 T: alpha-1-antitrypsin deficiency.

**HCC**	**N**	**Male gender**	**Age**	**Viral hepatitis**	**Alcohol**	**Viral hepatitis + alcohol**	**Cryptogenic**	**Alpha 1 T**
Not encapsulated	10	100%	53.9±8.3	3	3	2	1	1
Encapsulated	10	70%	53.6±7.7	7	2	0	1	0

References

1. Govaere O, Komuta M, Berkers J, Spee B, Janssen C, de Luca F, et al. Keratin 19: a key role player in the invasion of human hepatocellular carcinomas. Gut. 2014; 63: 674-85.
2. Borghesan M, Fusilli C, Rappa F, Panebianco C, Rizzo G, Oben JA, et al. DNA Hypomethylation and Histone Variant macroH2A1 Synergistically Attenuate Chemotherapy-Induced Senescence to Promote Hepatocellular Carcinoma Progression. Cancer research. 2016; 76: 594-606.
3. Rappa F, Greco A, Podrini C, Cappello F, Foti M, Bourgoin L, et al. Immunopositivity for histone macroH2A1 isoforms marks steatosis-associated hepatocellular carcinoma. PloS one. 2013; 8: e54458.
